# Ultrasensitive Chemiresistive Gas Sensors Based on Dual-Mesoporous Zinc Stannate Composites for Room Temperature Rice Quality Monitoring

**DOI:** 10.1007/s40820-024-01645-5

**Published:** 2025-01-24

**Authors:** Jinyong Xu, Xuxiong Fan, Kaichun Xu, Kaidi Wu, Hanlin Liao, Chao Zhang

**Affiliations:** 1 College of Mechanical Engineering, Yangzhou University, Yangzhou, 225127 People’s Republic of China; 2 ICB UMR 6303, CNRS, Univ. Bourgogne Franche-Comté, UTBM, 90010 Belfort, France

**Keywords:** Zinc stannate, Semiconductors, Dual-mesoporous structure, Gas sensor, Biomarker sensing

## Abstract

The integration of dual-mesoporous structures, the construction of heterojunctions, and the incorporation of highly concentrated oxygen vacancies are pivotal for advancing metal oxide-based gas sensors. Nonetheless, achieving an optimal design that simultaneously combines mesoporous structures, precise heterojunction modulation, and controlled oxygen vacancies through a one-step process remains challenging. This study proposes an innovative method for fabricating zinc stannate semiconductors featuring dual-mesoporous structures and tunable oxygen vacancies via a direct solution precursor plasma spray technique. As a proof of concept, the resulting zinc stannate-based coatings are applied to detect 2-undecanone, a key biomarker for rice aging. Remarkably, the zinc oxide/zinc stannate heterojunctions with a well-defined secondary pore structure exhibit exceptional gas-sensing performance for 2-undecanone at room temperature. Furthermore, practical experiments indicate that the developed sensor effectively identifies adulteration in various rice varieties. These results underscore the potential of this method for designing metal oxides with tailored properties for high-performance gas sensors. The enhanced adsorption capacity and dual-mesoporous features of this semiconductor make it a promising candidate for sensing applications in agricultural food safety inspections.

**Supplementary Information:**

The online version contains supplementary material available at 10.1007/s40820-024-01645-5.

## Highlights

- Dual-mesoporous heterostructured semiconducting metal oxides were directly fabricated using a simple template-free method, optimizing porosity and improving surface area for enhanced gas-sensing.
- The fabricated sensor exhibited high sensitivity (11.03 for 13 ppm), a rapid response time (21 s), and an impressively low theoretical detection limit (431 ppb) for 2-Undecanone at room temperature.
- An innovative real-time method was developed for analyzing characteristic biomarkers of rice aging, enabling accurate and timely monitoring of rice quality.

**Supplementary Information:**

The online version contains supplementary material available at 10.1007/s40820-024-01645-5.

## Graphical abstract

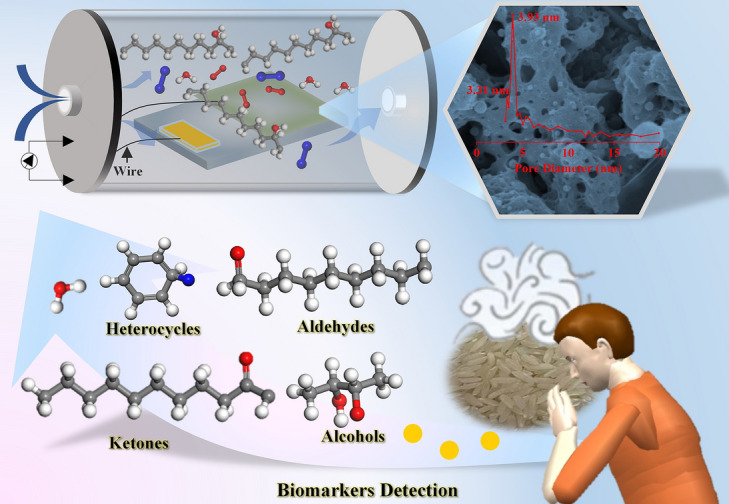

## Introduction

Rice is well supplied with numerous nutrients including carbohydrates, lipids, and proteins. With changes in the storage surroundings (i.e., temperature, pressure, humidity, etc.), these nutrients can deteriorate under enzyme activities in rice. This deterioration leads to the formation of numerous volatile organic compounds (VOCs), which are highly associated with the aging of rice. Among those VOCs, 2-undecanone stands out as a ketone derived from the oxidative breakdown of unsaturated fatty acids in rice [1–3]. The concentration of 2-undecanone increases with the oxidation rate of fatty acids during aging, making it a valuable biomarker for assessing rice quality. Currently, techniques such as headspace solid-phase micro-extraction combined with gas chromatography-mass spectroscopy and near-infrared spectroscopy have been extensively available for quantifying 2-undecanone in aged rice [4]. Despite their high stability and precision, these methods face challenges such as complex procedures, limited qualitative capabilities, and lengthy testing times. These limitations hinder their widespread application for rice quality inspection. Therefore, there is a significant need for a real-time and non-destructive method for monitoring 2-undecanone, offering exceptional sensitivity, selectivity, and long-term stability.

The development of gas sensors based on semiconducting metal oxides for VOC monitoring has gained prominence due to their cost-effective fabrication, superior physicochemical stability, and excellent sensing properties. However, most metal oxides require elevated operating temperature (above 200 °C) owing to their wide bandgap, which can exceed the boiling point of 2-undecanone and potentially lead to its decomposition through pyrolysis. Accordingly, the utilization of metal oxides for 2-undecanone sensing has not been explored to date. Among those metal oxides, zinc stannate stands out as a promising candidate for VOC sensing applications. It boasts high electron mobility (~ 10–15 cm^2^ V^−1^ S^−1^), significant electrical conductivity (~ 10^4^ S cm^−1^), and low light visible adsorption (bandgap ~ 3.1–3.9 eV) [5]. Given these characteristics, metal oxides with unique morphologies and heterostructures are highly desired for gas-sensing. Zinc stannate can be fabricated into various structures, such as porous, hierarchical, and core–shell configurations. For instance, Cao et al. [6] prepared mesoporous zinc stannate (ZnSnO_3_) thin films using magnetron sputtering, which demonstrated a high sensitivity of 11.5–50 ppm ethanol at 290 °C, attributed to the large specific surface areas of the mesoporous structures. Recent interest has focused on metal oxides with dual-mesoporous structures, as they offer enhanced pore connectivity compared to single-mesoporous structures, significantly improving gas molecules transmission through Knudsen diffusion and interaction with active absorption sites. Ma et al. [7] synthesized transition metal oxides with dual-mesoporous structures using a co-assembly approach, achieving excellent gas-sensing performance with a sensitivity of 1.4 at an ultra-low concentration of 50 ppb for 3-hydroxy-2-butanone, surpassing previous sensor technologies. Furthermore, dual-mesoporous structures can prevent pore blockage caused by particle migration, enhancing their suitability for gas–solid interface reactions. Nevertheless, current methods for creating secondary pores in porous materials involve complex procedures, including using silica, polymers, or surfactants as templates. In light of these challenges, a more general and convenient route for fabricating dual-mesoporous zinc stannate is proposed.

Heterojunction construction is another effective strategy for enhancing the gas-sensing performance of metal oxides. Wang et al. [8] demonstrated that decorating ZnSnO_3_-based sensors with nano-TiO_2_ can lower their operating temperature. This improvement is attributed to the introduction of impurity energy bands, which create large band tails at the bottom of the conduction band, thereby reducing the bandgap. Consequently, the sensor requires less activation energy to excite electrons into the conduction band when heterojunctions are formed between TiO_2_ and ZnSnO_3_. Besides, modulating the electronic structure and surface chemical state of zinc stannate, such as through the incorporation of oxygen vacancy, is a highly effective approach for developing high-performance gas sensors. The formation of oxygen vacancies decreases the bandgap [9], facilitating electron transfer, which in turn lowers the operating temperature and enhances sensitivity. Wang et al. [10] synthesized hollow ZnSnO_3_ cubes with controllable oxygen vacancies using a simple hydrothermal route combined with alkali etching. Their results showed that ZnSnO_3_ with an oxygen vacancy ratio of 21.1% exhibited significantly improved gas-sensing properties compared to unetched ZnSnO_3_ nanocubes. Specifically, the operating temperature decreased by 12%, and sensitivity increased by 400%. In summary, combining heterojunction construction with oxygen vacancy incorporation presents a promising approach to optimize the performance of zinc stannate sensors for detecting 2-undecanone. Nevertheless, this approach typically involves multiple steps: first, preparing the heterojunctions via various chemical synthesis methods, and then treating the solid precipitates through processes such as annealing under controlled atmospheres or etching for different durations. These methods, however, still face limitations such as insufficient crystal defects and slow particle growth, which constrain further improvements in zinc stannate's gas-sensing performance.

Plasma spray, with its remarkable advantages including ultra-high temperatures (exceeding 10,000 K), extremely rapid cooling rates (10^5^–10^7^ K s^−1^), and a highly reducing atmosphere (H_2_), is an effective technique for fabricating mesoporous zinc stannate with concentrated oxygen vacancies. The distinct volatilization rates of Zn and Sn ions during the rapid cooling process lead to the formation of heterojunctions. Meanwhile, a small radius of the oxygen atom at the site hinders the formation of stable zinc stannate, making it more prone to forming heterojunctions under high-temperature conditions. The reducing atmosphere further promotes the incorporation of highly concentrated oxygen vacancies on the surface of zinc stannate-based gas-sensing materials. In this work, high-performance gas sensors are directly fabricated by integrating dual-mesoporous structures, heterojunction construction, and controlled concentrations of oxygen vacancy into ZnO, SnO_2_, and ZnO/ZnSnO_3_ using solution precursor plasma spray (SPPS) with the corresponding precursors. A thorough investigation is conducted to evaluate their room temperature gas-sensing performance towards ppm-level 2-undecanone. In addition, the practical application of these sensors in assessing rice quality is examined with two different types of rice stored for varying periods. This study offers novel insights into the rapid construction of heterojunctions and the regulation of dual-mesoporous structures in metal oxides, offering valuable guidance for the development of high-performance electronic noses for agricultural food safety inspection. Additionally, the fabrication of semiconductor-based gas sensors presents an opportunity to explore gas-sensing reactivity at the molecular level.

## Experimental Section

### Plasma Spraying Deposition

**Fig. 1:**
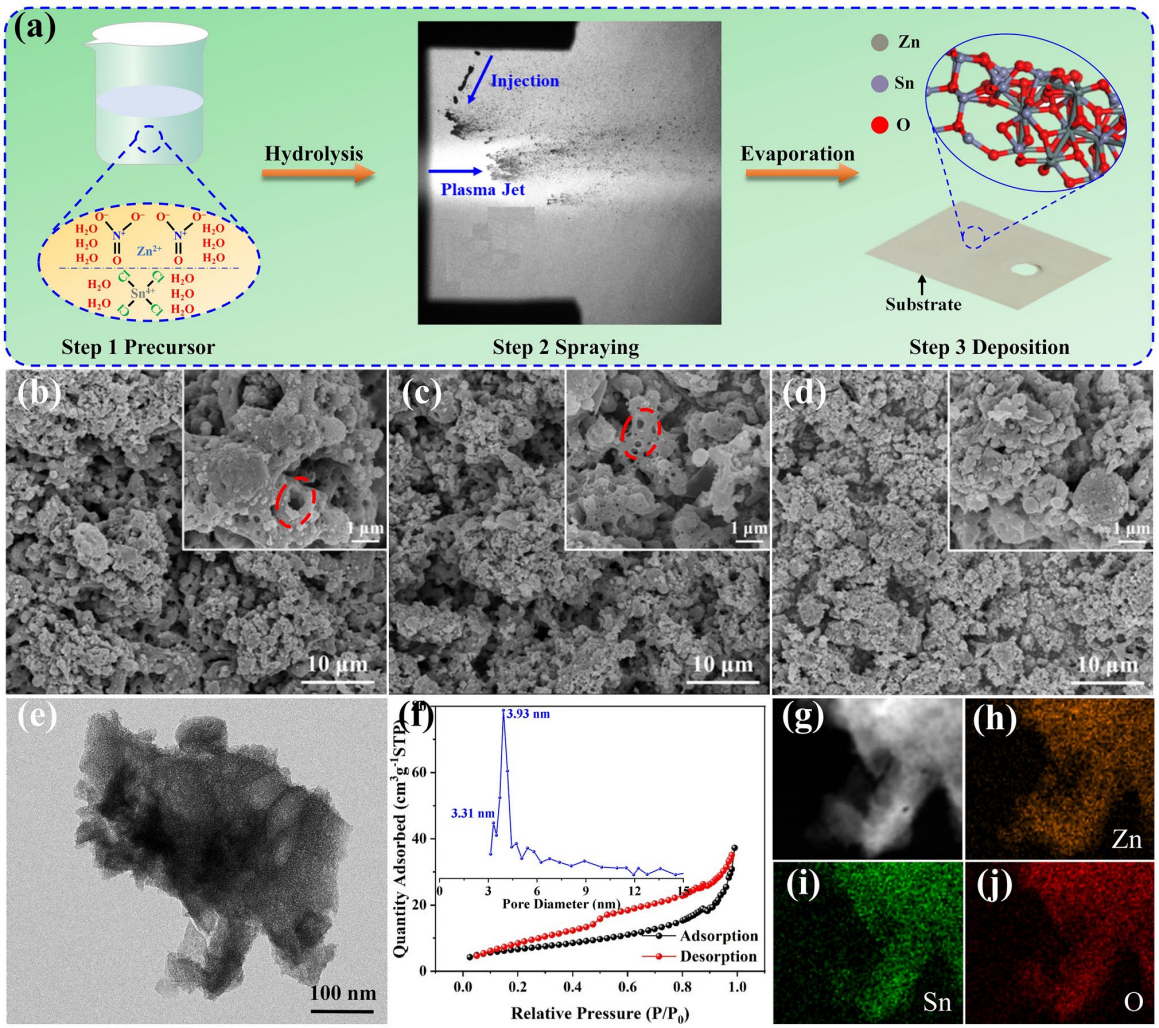
**a** Schematic illustration of the preparation process. **b-d** FE-SEM images of H1, H3, and H5. **e** TEM image and the corresponding size distribution of H3. **f** Nitrogen adsorption–desorption isotherm and the corresponding pore diameter distribution curve of H3. **g** High-angle annular dark-field scanning transmission electron microscope image and **h-j** corresponding EDS elemental mapping distributions in H3

Zinc nitrate hexahydrate (Zn(NO_3_)_2_∙6H_2_O) and tin chloride pentahydrate (SnCl_4_∙5H_2_O) were used as precursors. These inorganic precursors, in a 1:1 molar ratio, were dissolved in deionized water to create saturated aqueous solutions with a concentration of 0.3 M under continuous stirring. Plasma spraying deposition was carried out using a plasma torch (F4, Oerlikon Metco, Switzerland) mounted on a six-axis robotic arm (ABB, Switzerland). This setup allowed for the deposition of nanocrystals onto commercial alumina oxide plates with dimensions of 22 × 28 × 1 mm^3^. As shown in Figs. 1a and S1, the precursor solutions were injected into the plasma jet using a stainless steel needle, with pressurized nitrogen serving as the driving gas. The detailed SPPS parameters are shown in Table S1. To achieve rational design of mesoporous nanostructures and precise incorporation of highly concentrated oxygen vacancies, the flow rate of the secondary plasma gas (hydrogen) was varied between 1, 3, and 5 L min^−1^. The resulting samples were labeled H1, H3, and H5, respectively. For comparative analysis, gas sensors based on pure ZnO and SnO_2_ were also fabricated using the corresponding precursors.

### Characterization

The morphology and structure of the obtained materials were characterized using a field emission-scanning electron microscope (FE-SEM, S4800, Japan) and transmission electron microscopy (TEM, Tecnai 12, Netherlands). Phase compositions were determined by X-ray diffraction (XRD) with a Bruker D8 Advance instrument, utilizing Cu-Kα radiation (*λ* = 1.5408 Å). Elemental distribution within the samples was analyzed using an energy-dispersive X-ray spectroscope (EDS) attached to the TEM. Ultraviolet–visible spectroscopy (UV–Vis) absorption spectra were recorded with a Cary 5000 spectrometer (USA) across the wavelength range of 200–800 nm. Surface chemical states were examined via X-ray photoelectron spectroscope (XPS) using an ESCALAB 250Xi system. The specific surface areas and pore sizes of the samples were evaluated using the Brunauer–Emmett–Teller (BET) models.

### Measurement of Sensors

The gas-sensing measurements in this experiment followed the procedure outlined in Ref. [11]. A schematic illustration of the gas-sensing testing setup is shown in Fig. S2. Briefly, air was bubbled through 2-undecanone, and the resulting target gas was then diluted before interacting with the sensing materials. The concentration of 2-undecanone was calculated using Eq. 1 and could be adjusted by varying the flow rates of the mass flow controllers (MFCs), as detailed in supplementary material. The resistances of the sensor in air (*R*_*a*_) and in the presence of guest molecules (*R*_*g*_) were recorded in real-time using a homemade gas tester (HCRK-SD101, China). Sensitivity was defined as *R*_*a*_*/R*_*g*_ for reducing gas or *R*_*g*_*/R*_*a*_ for oxidizing gas. The response time (*τ*_*res*_) and recovery time (*τ*_*rec*_) were defined as the time required to reach 90% of the saturation value after introducing or removal of the target gas.

1 $${\text{Concentration}}\,\left( {{\text{ppm}}} \right)\, = \,\left( {{1}0^{{6}} \, \times \,{\text{Vapor}}\,{\text{pressure}}} \right)/{76}0$$

Where the vapor pressure of 2-undecanone is 0.1 mmHg at 25 °C.

## Results and Discussion

Figure 1 illustrates the deposition process and structural analysis of zinc stannate heterojunctions. As shown in Fig. 1a, uniform transparent solutions containing Zn(NO_3_)_2_∙6H_2_O and SnCl_4_∙5H_2_O precursors in a 1:1 molar ratio were successfully injected into the plasma jet, as confirmed by SprayCam equipment, under the liquid parameters shown in Table S1. The molten droplets condensed on the substrate surface, forming gas-sensing coatings with varying thicknesses and surface uniformity. The formation process involves several stages: evaporation, precipitation, pyrolysis, and melting. The cross-sectional profiles reveal that the thickness of H1 was measured at 20.08 ± 1.47 μm, making it the thickest among the samples (10.89 ± 1.02 μm for H3, 7.48 ± 0.81 μm for H5), with a higher surface roughness, as vividly shown in Fig. S3. This variation is primarily associated with the hydrogen flow’s impact on the plasma jet’s energy density, which in turn affects the efficiency of the precursors entering the plasma jet. As a result, the thickness and the surface roughness of the coatings deposited by SPPS can be effectively regulated by adjusting the hydrogen flow, thereby affecting the gas-sensing performance [12]. Figure 1b–d presents SEM images that reveal the morphologies of the samples. At hydrogen flows of 1 and 3 L min^−1^, numerous nanometer-sized pores are visible on the coating surface, displaying a highly porous structure. In contrast, H5 exhibits a denser structure. This difference may be attributed to the substantial increase in the plasma jet’s energy density at higher hydrogen flow, which propels molten droplets toward the substrate with greater energy and velocity, leading to the formation of flattened nanoparticles that are tightly connected to form a dense coating. The nanoporous structures observed in the FE-SEM images are particularly notable for providing numerous active sites and shorter diffusion distances, which are advantageous for electronic transmission and guest molecular diffusion, ultimately improving sensitivity and reducing response time [13, 14]. Furthermore, Figs. 1e and S5 show the TEM images of H1, H3, and H5, which reveals that all samples comprise various nanoparticles, consistent with the FE-SEM results. The nitrogen adsorption–desorption isotherms and pore size distributions shown in Figs. 1f and S4 reveal that the materials H1 and H3 exhibit a dual-mesoporous structure. Notably, H1 has a secondary pore centered at 2.97 nm in addition to a main mesopore distribution at 3.93 nm. The dual-mesoporous structure primarily arises from the combined effects of solvent evaporation, gas release during the precursor’s transformation in the plasma spraying process, and the self-assembly behavior of the precursor particles. Specifically, the large mesopores are formed due to rapid solvent evaporation and gas evolution in the initial stages of the spraying process, while the smaller mesopores result from slower diffusion and solidification of the precursor at later deposition stages. At low hydrogen flows (1 and 3 L min^−1^), the temperature and velocity of the droplets are moderate, creating a controlled environment that allows these mechanisms to occur, thereby facilitating the formation of the dual-mesoporous structure. In contrast, at a high hydrogen flow (5 L min^−1^), the increased temperature and velocity accelerate solidification and disrupt the balance required for gas release and precursor self-assembly. This rapid solidification and increased turbulence prevent the development of the hierarchical pore architecture, resulting in the disappearance of the dual-mesoporous structure. Table S3 summarizes the textural properties of all samples, revealing that increasing hydrogen flow decreases the size distribution of the secondary pore. These findings strongly suggest that hydrogen flow significantly influences the tuning of the dual-mesoporous structure in zinc stannate. In addition, the EDS elemental mapping distributions shown in Figs. 1h–j and S6 confirm that the material consists of Zn, Sn, and O, uniformly distributed along the sample’s contour, indirectly verifying the successful deposition of gas-sensing coatings via SPPS.

**Fig. 2:**
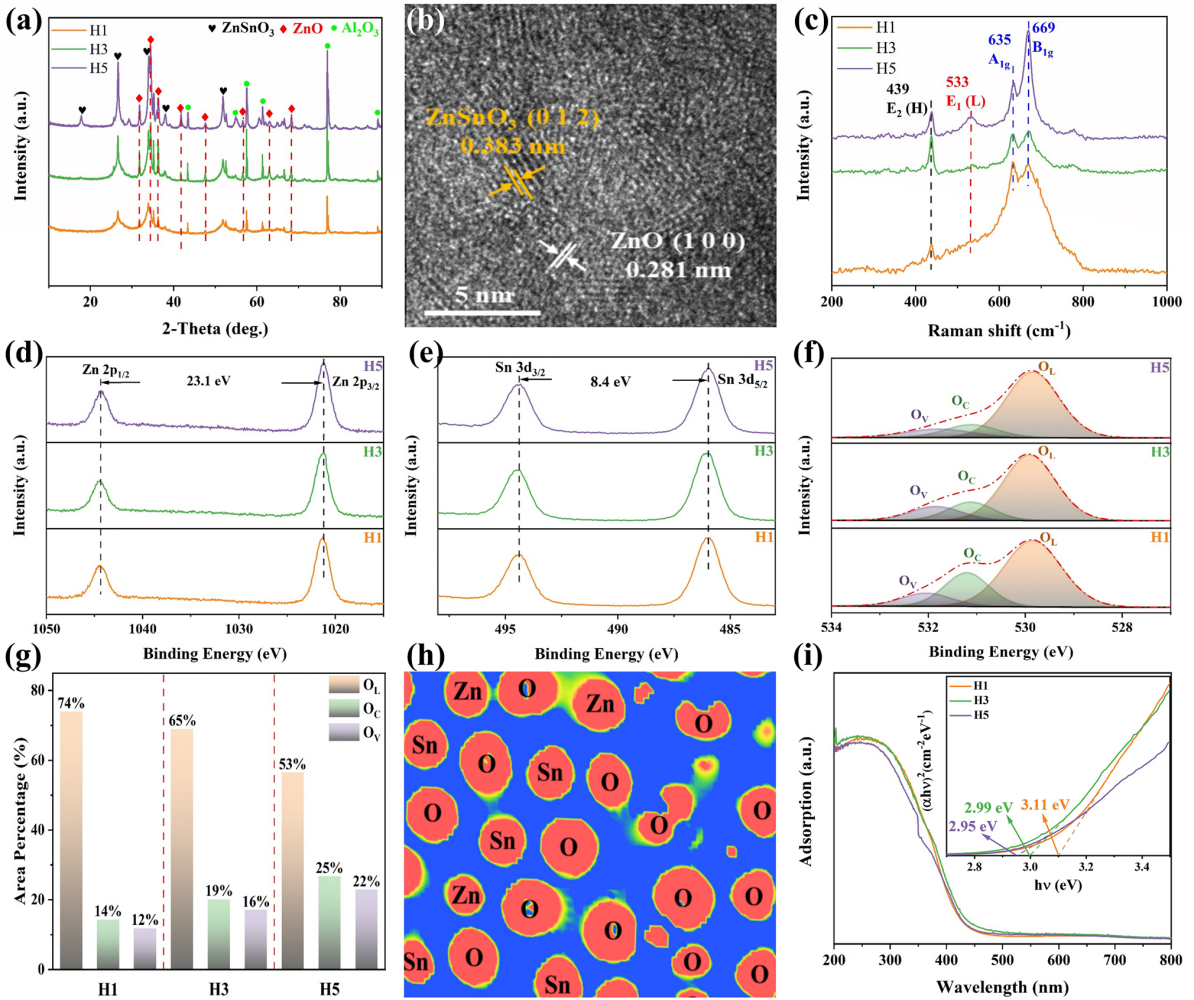
**a** XRD patterns of the samples. **b** HRTEM image of H3. **c** Raman spectra of the three samples. **d-f** High-resolution XPS spectra of all samples for Zn, Sn, and O elements, respectively. **g** Results of the curve fitting of the O 1*s* spectra of the three samples. **h** Charge distributions of zinc stannate heterojunctions in a two-dimensional plane. **i** UV–Vis absorption spectra and the corresponding Tauc curves of the samples

To give insight into the phase compositions of the samples deposited by SPPS, XRD analysis of H1, H3, and H5 is conducted, as shown in Fig. 2a. Besides the presence of Al_2_O_3_ phases (substrate) (Fig. S7), the diffraction peaks match well with the standard card of ZnSnO_3_ (JCPDS No. 28-1486), where the three distinct peaks centered at 26.50°, 33.79°, and 51.59° correspond to the (012), (110), and (118) crystal planes, respectively [9]. There is no significant shift in the diffraction peaks. Besides, several characteristic peaks at 2θ of 31.72°, 34.40°, 36.21°, 47.49°, 56.51°, and 62.80° are attributed to the (100), (002), (101), (102), (110), and (103) crystal planes, respectively. These are consistent with the hexagonal wurtzite structure of ZnO (JCPDS No. 36-1451) [15], indicating that all samples consist of both ZnO and ZnSnO_3_ phases. Furthermore, the ZnO (100) plane with an interplanar spacing of 0.281 nm can be observed in the HRTEM image shown in Fig. 2b, further confirming the formation of heterojunctions between ZnO and ZnSnO_3_ interfaces, which may be related to the different volatilization rates of Zn and Sn ions during spraying. This demonstrates that SPPS is a novel and efficient route for constructing heterostructures, especially in ternary metal oxides. Additionally, the circular dot arrays observed in the selected area electron diffraction (SAED) pattern in Fig. S8 display the polycrystalline characteristics of the gas-sensing coating deposited by SPPS, and the lattice spacing on the diffraction rings aligns well with the XRD pattern. Interestingly, as evident from the XRD patterns, the intensity of the characteristic peaks becomes stronger with the increase in hydrogen flow, indicating a higher degree of crystallization in H5 due to the crystallite growth (Table S2). Raman spectroscopy is employed to inspect the occurrence of local structural changes and examine the defects including oxygen vacancy caused by the loss of symmetry and reorientation. The Raman spectra, shown in Fig. 2c illustrate four vibrational modes: E_2_ (H), E_1_ (L), A_1g_, and B_1g_ at 439, 533, 635, and 669 cm^−1^, respectively. More specifically, the E_2_ (H) mode is indicative of the hexagonal wurtzite structure of ZnO, reflecting oxygen motions and sensitivity to internal stress. The Raman shifts at 635 and 669 cm^−1^ are attributed to ZnSnO_3_ and are associated with the stretching vibrations of short Zn–O–Zn bonds [16]. Notably, the intensity of the E_1_ (L) mode at 533 cm^−1^ increases with the hydrogen flow, suggesting an elevated relative concentration of oxygen vacancy [17]. These results consistently demonstrate that highly concentrated defects are generated during the spraying process as the hydrogen flow varies.

The chemical states of various elements and the effects of cation substitution on oxygen species absorption were investigated using XPS analysis, which revealed the core level peaks for Zn 2*p*, Sn 3*d*, and O 1*s*. The survey spectra shown in Fig. S9 show the co-existence of Zn, Sn, and O, which aligns with the EDS mapping illustrated in Fig. 1h–j. The binding energy shifts of Zn and Sn observed in the XPS spectra indicate changes in their chemical environments. In Fig. 2d, the peaks at 1044.8 and 1021.7 eV correspond to Zn 2*p*_1/2_ and Zn 2*p*_3/2_, respectively. This shift is attributed to interactions with oxygen atoms, which alter the local electronic density around Zn ions due to coordination effects with neighboring atoms. The spin–orbit splitting value of 23.1 eV confirms that Zn exists in the + 2 oxidation state [18]. Similarly, the Sn 3*d* spectrum in Fig. 2e reveals a spin–orbit splitting of 8.4 eV between Sn 3*d*_3/2_ and Sn 3*d*_5/2_, reflecting changes in the bonding environment of Sn. This analysis verifies that Sn atoms are in the + 4 valence state [19], consistent with the Zn 2*p* results. These binding energy shifts confirm the formation of a robust zinc stannate structure, highlighting the strong interactions between Zn, Sn, and O that contribute to the stability and functionality of the material. Additionally, the O 1*s* spectra, illustrated in Fig. 2f, are deconvoluted into three peaks at 529.6, 531.5, and 532.3 eV, which are assigned to lattice oxygen (O_L_), chemisorbed oxygen (O_C_), and oxygen vacancy (O_V_), respectively [20]. Figure 2g summarizes the quantification of the O 1*s* spectra. Notably, there is a significant variation in the relative proportions of O_C_ and O_V_ components among the three samples. Impressively, the area percentages of O_C_ and O_V_ in H5 are higher than those in the other two samples, which is closely related to the energy density of the plasma jet supported by a higher hydrogen flow. The EPR spectrum results shown in Fig. S10 also indicate that the signal of H5 is the highest, which proves that the concentration of O_V_ in H5 is the highest among the three samples. The improvement in the relative proportions of these oxygen species is the core component of sensing coatings to directly interact with the target gas [21]. Moreover, a two-dimensional plane’s charge density distribution analysis (Fig. 2h) was performed to disclose the electronic configuration of zinc stannate heterojunctions [22]. This analysis indicates that the valence and conduction bands are primarily composed of the 1*s* orbitals from oxygen (O), the 2*p* orbits from zinc (Zn), and the 3*d* orbits from tin (Sn). To explore the possibility of the developed gas sensor functioning at low operating temperatures, the UV–Vis spectra of the three samples were analyzed, as shown in Fig. 2i. Absorption tails in both visible and near-infrared regions are observed, related to free electrons and polarons induced by oxygen vacancy [23]. Using the Tauc formula, the bandgap energies (*E*_*g*_) of H1, H3, and H5 are estimated to be 3.11, 2.99, and 2.95 eV, respectively, as determined by the intercept of the tangent line to the X-axis. These values confirm that the samples belong to the category of wide bandgap semiconductors, facilitating the fabrication of devices with improved gas-sensing capabilities at relatively low operating temperatures.

**Fig. 3:**
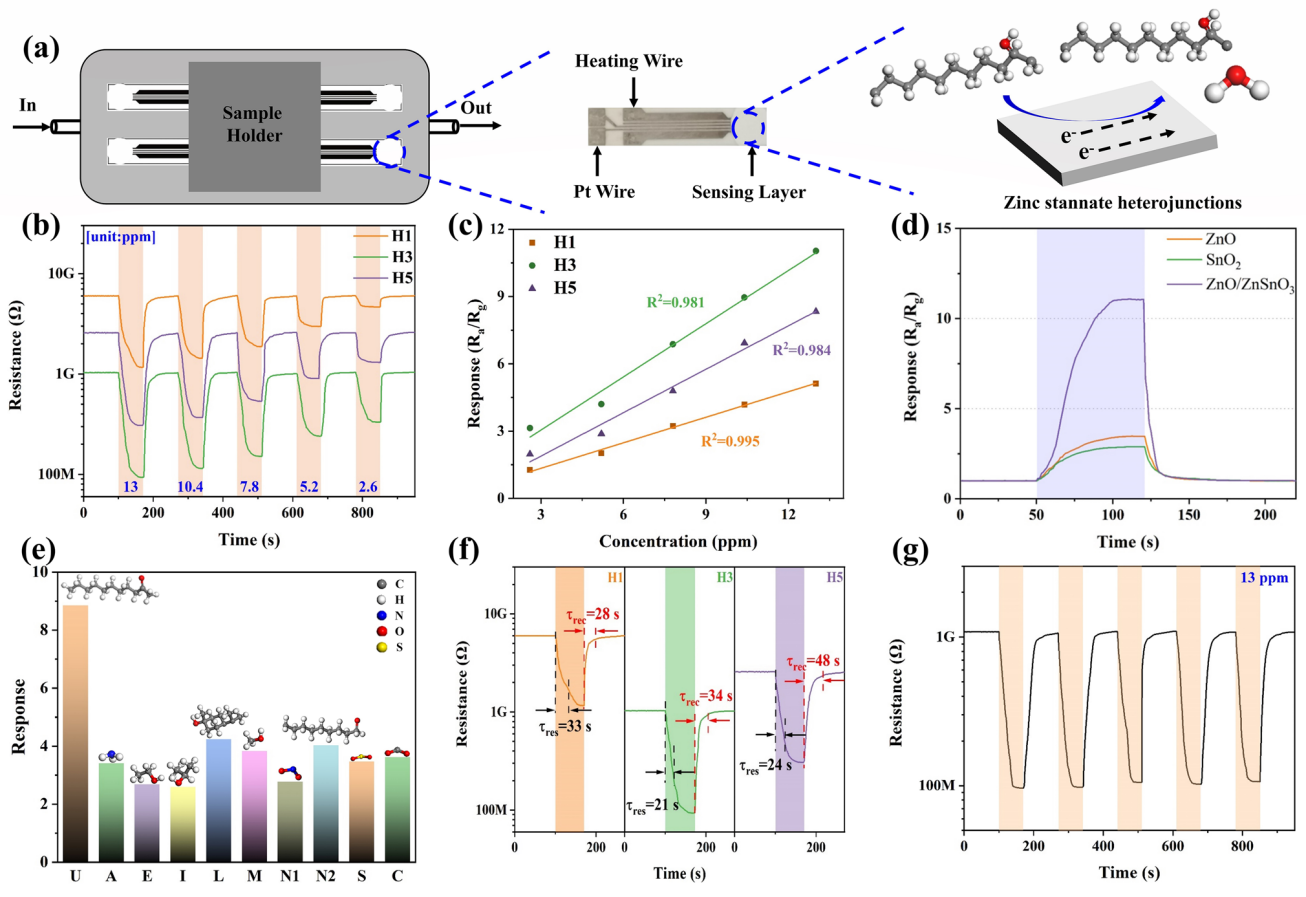
**a** Sketch and optical photographs of zinc stannate-based gas sensors. **b** Changes in the electrical resistance of the sensors towards 2-undecanone with a concentration ranging from 2.6 to 13 ppm. **c** Response of all sensors versus gradient 2-undecanone concentrations. **d** Responses of gas sensors based on ZnO, SnO_2_, and ZnO/ZnSnO_3_ fabricated with a hydrogen flow of 3 L min^−1^ towards 13 ppm 2-undecaone. **e** Responses of the H3-based gas sensor towards different gases (U: 2-undecanone, A: ammonia, E: ethanol, I: isopropanol, L: linalool, M: methanol, N1: NO_2_, N2: nonanal, S: SO_2_ (100 ppm), C: CO_2_ (400 ppm), all gas concentrations are at 10 ppm excluding notation). **f** Response-recovery curves of all sensors towards 13 ppm 2-undecanone. **g** Repeated response-recovery curves of the H3-based gas sensor towards 13 ppm 2-undecanone

To investigate the gas-sensing properties, precursors were directly deposited onto ceramic plates with Pt electrodes, leading to the fabrication of miniaturized semiconductor gas sensors. The gas-sensing characteristics of all materials were then examined using a platform that utilizes side-heated gas sensors, as shown in Fig. 3a. The sensing materials adsorb 2-undecanone, a biomarker for rice aging, which is then oxidized by the chemisorbed oxygen species, with electrons transferring from 2-undecanone to ZnO/ZnSnO_3_ heterojunctions. The semiconductor characteristics and gas-sensing ability of heterojunctions, as mentioned above, can be achieved at low working temperatures under the activation of oxygen vacancies. Accordingly, the gas-sensing characteristics of the samples were investigated under ambient conditions. Figure 3b shows the resistance changes of all sensors in response to varying concentrations of 2-undecanone, and their room temperature sensing attributes (i.e., sensitivity, τ_res_, and τ_rec_, etc.) are shown in Table S4. Upon exposure to the carrier gas (79%N_2_+21%O_2_), the sensor’s resistance rapidly increases, with the baseline resistance (*R*_*a*_) ultimately reaching high values between 1 and 10 GΩ. When the target gas (2-undecanone) is exposed, the resistance decreases, and it returns to *R*_*a*_ after switching off the guest molecules, suggesting that all sensors display n-type semiconductor properties.

The resistance variations shown in Fig. 3b reveal a linear relationship between sensor response and gas concentration, as shown in Fig. 3c. As the gas concentration increases, the sensitivity of all sensors increases proportionally, indicating a positive linear relationship between sensitivity and concentration. This characteristic makes these sensors particularly suitable for the quantitative analysis of 2-undecanone in practical gas-sensing applications. The slops for the sensitivity of H1, H3, and H5 are 0.38 (y = 0.38x + 0.21), 0.79 (y = 0.79x + 0.68), and 0.65 (y = 0.65x-0.05), with corresponding linear fitting coefficients (*R*^*2*^) of 0.995, 0.981, and 0.984, respectively. Among them, H3 exhibits the highest sensitivity, reaching 11.03 towards 13 ppm 2-undecanone, with a theoretical detection limit of 431 ppb calculated based on the equations from Ref. [24]. Interestingly, the improvement in sensitivity aligns closely with the relative concentrations of oxygen species (O_C_ and O_V_) measured by XPS, which contributes to the enhanced sensitivity. Nevertheless, compared to H3, H5 displays a denser structure (Fig. 1d), limiting the pathways available for gas molecule permeability, thereby reducing sensitivity. As shown in Fig. S12, the sensor response initially increased with higher hydrogen flow rates but decreased beyond a certain threshold. This reduction in response is closely linked to the interplay between coating thickness and grain size. At higher hydrogen flow rates, such as in the H5 sample, the reduced coating thickness contributed to a significant increase in the average crystallite size, as shown in Table S2. However, this increase led to a mismatch between the crystallite size and twice the Debye length [25], which adversely affected the sensor’s charge carrier dynamics. Inspired by this, apart from incorporating a large number of O_V_, designing dual-mesoporous heterojunctions with an optimal thickness is considered the most effective tactic for boosting the sensitivity of gas sensors based on these semiconductors.

Figure 3d presents the sensitivity of gas sensors based on ZnO, SnO_2_, and ZnO/ZnSnO_3_, all deposited with a hydrogen flow of 3 L min^−1^, towards 13 ppm of 2-undecanone. Among these, the ZnO/ZnSnO_3_-based gas sensor displays the highest sensitivity. This improvement is chiefly attributed to the intrinsic property of semiconductors and the formation of heterojunctions. Besides, as shown in Fig. S11, the *R*_*a*_ values of the gas sensors based on H1, H3, and H5 are approximately 6.01, 1.03, and 2.56 GΩ, respectively, when operating at room temperature. The incorporation of a significant number of oxygen vacancies contributes to a decrease in *R*_*a*_, followed by an increase when the hydrogen flow is set at 5 L min^−1^. The effects of oxygen vacancies are particularly noteworthy here, as electrons trapped in the donor level can be excited into the semiconductor’s conduction band, thereby increasing the carrier concentration and ultimately resulting in lower resistance (*R*_*a*_) [26]. Moreover, the observed increase in *R*_*a*_ of H5 might be attributed to its reduced thickness (Fig. S3). It is reported that a reduction in thickness leads to a larger crystallite size in semiconductors, which, in turn, decreases the grain boundary areas. Kou et al. [27] also reported that the baseline resistance of metal oxide-based gas sensors tends to increase as the crystallite sizes significantly decrease. The XRD characterization data further supports this observation, with average crystallite sizes for H1, H3, and H5 calculated as 7.53, 10.36, and 14.43 nm, respectively (Table S2).

Additionally, the electrical noise of all sensors is analyzed by measuring the average resistance fluctuation as purified air is introduced. Figure S13 illustrates the noise levels for the gas sensors, which are around 0.135%, 0.024%, and 0.091% for H1, H3, and H5, respectively. H3 exhibits the lowest noise level, likely due to its low baseline resistance (*R*_*a*_) [28]. Selectivity, a key parameter for estimating the performance of gas sensors, is greatly influenced by factors such as adsorption ability, reaction rate, and the intrinsic characteristics of guest molecules. Herein, nine varieties of gases including ammonia, ethanol, isopropanol, linalool, methanol, nonanal, NO_2_, SO_2_, and CO_2_ were selected to be interfering gases. The sensitivity of the H3-based gas sensor towards 2-undecanone is at least twice that of other interfering gases, as shown in Fig. 3e. This superior selectivity is attributed to H3’s higher adsorption ability and stronger reducibility for the target gas, leading to faster reaction rates and more efficient electron transfer compared to other gases. Besides, the τ_res_ and τ_rec_ for sensors exposed to 13 ppm of 2-undecanone are calculated. For H1, H3, and H5, τ_res_ and τ_rec_ values are 33/28, 21/34, and 24/48 s, respectively, indicating rapid response/recovery times for all sensors (Fig. 3f). These results are closely related to their nanoscale dual-mesoporous structures. The repeatability of H3 was assessed by its response-recovery curve over five cycles (Fig. 3g), showing minimal fluctuations in sensitivity and *R*_*a*_, with deviations of 10.7% and 2.8%, respectively. The sensor maintains nearly constant sensitivity over a 30-day testing period, indicating outstanding long-term stability (Fig. S14). Regarding practical applications, the effect of humidity on sensor performance is also considered. The sensitivity and *R*_*a*_ of the H3-based gas sensor are measured under varying relative humidity (RH) levels (20%, 40%, 60%, and 80%) for 10 ppm 2-undecanone (Fig. S15). Sensitivity and *R*_*a*_ decrease by 59.6% and 51.1%, respectively, as RH increases from 0 to 40%. This is due to increased humidity causing the interaction between adsorbed oxygen and water molecules to form inactive hydroxyl groups, which negatively impacts the sensing interaction [29]. Meanwhile, 2-undecanone has the characteristic of being insoluble in water, which is another important factor in reducing the sensitivity. Despite this, the sensor maintains substantial sensitivity (*R*_a_/*R*_g_ = 2.09) even at 80%RH, likely due to the heterojunction structure, where one component has a strong affinity for water molecules, while the other is sensitive to 2-undecanone [30]. These results indicate that the dual-mesoporous ZnO/ZnSnO_3_ nanoscale heterojunction-based gas sensor manufactured by SPPS is effective for detecting ppm-level 2-undecanone across various humidity conditions. Future improvements should focus on incorporating functional organic materials on heterojunction surfaces, chemically tuning the sensing materials, and devising humidity compensation algorithms to substantially diminish the negative influence of moisture. Overall, dual-mesoporous zinc stannate semiconductors have proven to be promising semiconductors for developing sensing devices for 2-undecanone detection.

From the gas-sensing performance investigations, zinc stannate n–n nanoscale heterojunctions with dual-mesoporous structures show outstanding gas-sensing capabilities towards 2-undecanone at room temperature, including high sensitivity, rapid response times, and a low theoretical detection limit. The widely accepted gas-sensing mechanism of metal oxide semiconductors involves three primary steps: absorption, interaction, and desorption (Fig. 3b) [31]. As schematically shown in Fig. S17, in ZnO/ZnSnO_3_ composites, electrons transfer from ZnO to the ZnSnO_3_ surface due to differences in their calculated work functions and band gaps (7.116 and 1.22 eV for ZnSnO_3_, 4.691 and 0.74 eV for ZnO) (Fig. S16). This electron transfer causes the Fermi levels (*E*_*f*_) to shift towards more negative potentials until equilibrium is reached. When purified air is introduced, oxygen molecules capture free electrons from the conduction band, forming chemisorbed oxygen species (i.e., O_2_^−^, O^−^, O^2−^) depending on the sensor’s working temperature [32]. In this context, the gas-sensing experiments are conducted at room temperature, where O_2_^−^ is the predominant absorbed oxygen species. Simultaneously, the formation of accumulation layers on ZnSnO_3_ and electron depletion regions on ZnO results in reduced grain boundary barriers. This phenomenon enhances the gas-sensing properties of ZnO/ZnSnO_3_ heterojunctions by facilitating stronger interactions between guest molecules and the sensing materials. Although these interactions are challenging to investigate experimentally due to current limitations, they have been proposed in various studies [11, 19, 23, 33, 34]. In detail, when exposed to the reducing gas (2-undecanone), the gas is initially absorbed onto the sensor surface as gas molecules. The interaction between the adsorbed 2-undecanone (C_11_H_22_O) and O_2_^−^ leads to the generation of C_11_H_20_O (ad) (with a loss of H), H_2_O (g), and free electrons (e^−^), as schematically shown in Fig. S17b. During this sensing process, the trapped electrons are released into the conduction band of ZnO/ZnSnO_3_ n–n nanoscale heterojunctions, resulting in a decrease in the depletion layer thickness and the potential barrier height (Fig. S17). This ultimately causes a reduction in the sensor’s resistance.

The H3-based 2-undecanone sensor showcases remarkable gas-sensing characteristics, characterized by low operating temperature, high sensitivity, and rapid response time. These advantages arise from the combined effects of its dual-mesoporous nanostructures, which offer a high surface area (24.086 m^2^ g^−1^) and elevated relative proportions of oxygen species (19% for O_C_, and 16% for O_V_). This distinctive dual-mesoporous structure provides abundant active sites and significantly shorter diffusion paths, enhancing the adsorption and desorption of guest molecules [35, 36]. This, in turn, improves the sensor’s sensitivity and reduces response time. However, when the hydrogen flow increases to 5 L min^−1^, this unique dual-mesoporous structure gradually begins to degrade (Fig. S4), resulting in a marked reduction in both specific surface area (7.107 m^2^ g^−1^) and sensitivity (8.34 versus 13 ppm). Moreover, the area percentages of O_C_ and O_V_, as measured by XPS in H3 and H5, are higher than those in H1. This increase in oxygen species contributes to a greater adsorption capacity and faster reaction rate for 2-undecanone, thereby promoting electron transfer from the target gas to the sensing material’s surface, ultimately displaying enhanced gas-sensing performance. Most importantly, the thickness and surface roughness of the deposited gas-sensing coatings are crucial factors that must not be overlooked. Despite H5 having the highest relative proportions of O_C_ and O_V_ (Fig. 2g) and the narrowest bandgap (Fig. 2i), its thickness and surface uniformity are significantly decreased (Fig. S3). This reduction leads to increased contact resistance (Fig. 3b) and reduced specific surface areas (Table S3), limiting the number of active sites and pathways available for gas absorption and diffusion. Accordingly, in addition to precisely incorporating highly concentrated O_V_ and rationally designing dual-mesoporous structures, optimizing the thickness and surface uniformity of highly porous ZnO/ZnSnO_3_ n–n nanoscale heterojunction-based gas sensors is essential for enhancing gas-sensing characteristics, particularly for room temperature detection of 2-undecanone.

**Fig. 4:**
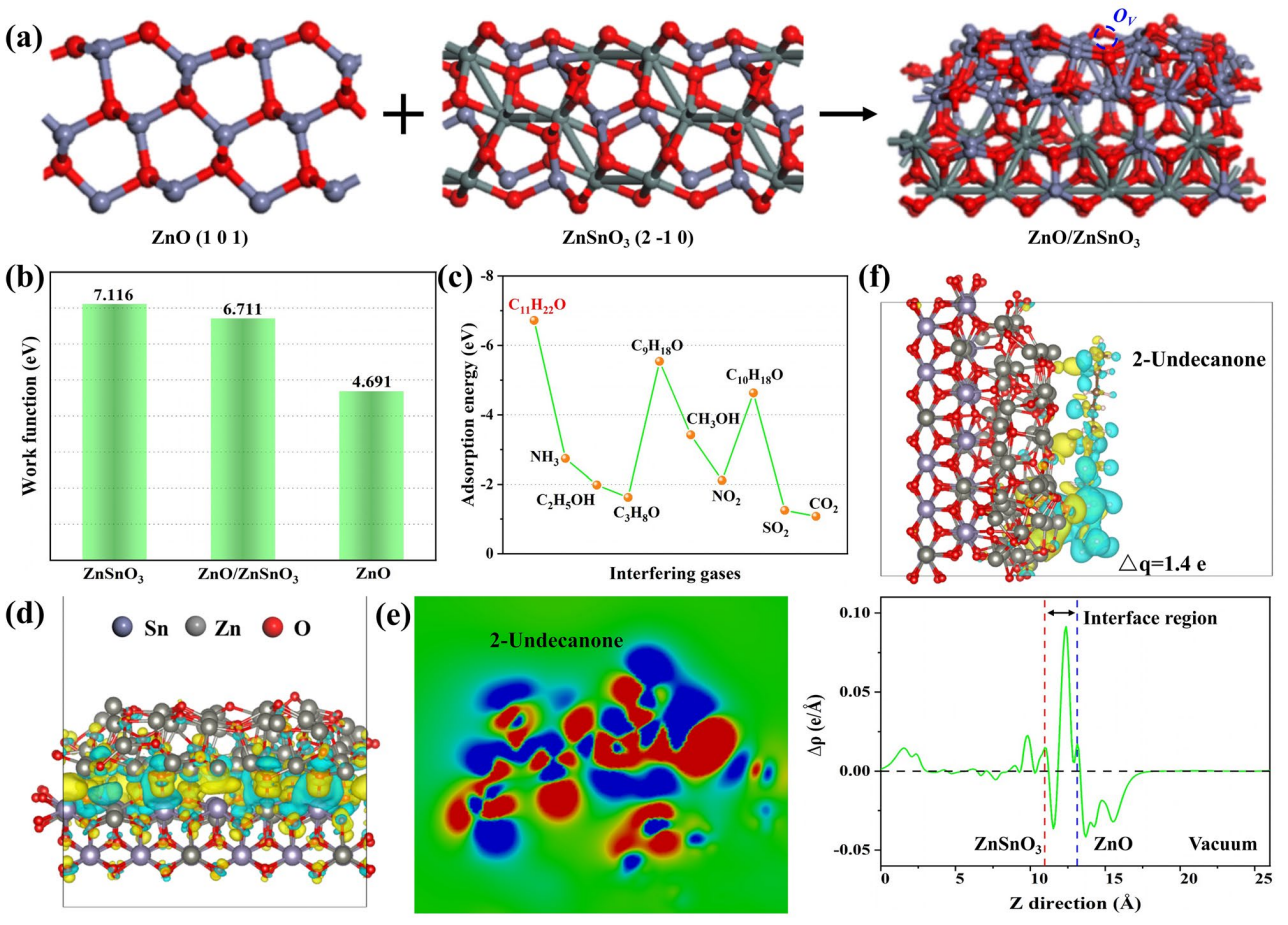
**a** View geometric structures of ZnO (101), ZnSnO_3_ (2–10), and ZnO/ZnSnO_3_ heterojunctions. **b** Calculated work functions of ZnSnO_3_, ZnO/ZnSnO_3_, and ZnO. **c** A comparison of adsorption energies for 10 gases on the surface of zinc stannate heterojunctions, low adsorption energy (further from zero) denotes stronger adsorption. **d** A three-dimensional view of the charge density differences of zinc stannate heterojunctions, in which the ZnSnO_3_ accumulated electron (yellow isosurfaces) while the ZnO depleted electron (blue isosurfaces) (The isosurface value is set as 0.0004 e Å^−3^). **e** Charge distribution in a two-dimensional plane after the adsorption of 2-undecanone. **f** A three-dimensional view of the charge density differences of zinc stannate (above) with the planar-averaged electron density difference along the Z direction (below) (The positive values denote electron accumulation, while the negative one denotes election depletion)

To explore the electron transfer mechanisms related to gas-sensing reactivity, density functional theory (DFT) calculations were conducted. Zinc stannate heterostructures were initially constructed and optimized to serve as a primary interface region for adsorbing 2-undecanone molecules (Fig. 4a and Table S5). Figure 4b illustrates the electrostatic potential at the Fermi level, which was used to explore the energy level alignment between ZnO and ZnSnO_3_, leveraging their work functions. The work functions of ZnSnO_3_ and ZnO layers are 7.116 and 4.691 eV, respectively (Fig. S16). For ZnO/ZnSnO_3_ heterojunctions, the work function is calculated to be 6.711 eV (Fig. S18). In this regard, the Fermi level shifts negatively towards the ZnSnO_3_ layer by 0.405 eV or positively towards the ZnO layer by 2.02 eV until an equilibrium Fermi level is reached at the interface, corroborating the previous gas-sensing mechanism analysis. As shown in Fig. 4c, the sensor exhibits the highest adsorption energy of −6.72 eV towards 2-undecanone among the nine interfering gases (with adsorption geometries shown in Fig. S19a–j), consistent with the gas-sensing performance shown in Fig. 3e. The calculated adsorption energy (*E*_*ads*_) consistently indicates the high selectivity of ZnO/ZnSnO_3_ heterojunctions for 2-undecanone molecules. Meanwhile, an *E*_*ads*_ value greater than 2.0 eV indicates that there may be a chemical adsorption process between the sensing materials and gas molecules, thus confirming the sensing process shown in Fig. S17. Overall, these DFT calculations provide robust theoretical support for the proposed room temperature gas-sensing mechanisms and underscore the superior selectivity of zinc stannate heterojunctions for detecting 2-undecanone molecules.

The redistribution of surface charges in heterojunctions significantly influences the electrical properties of the ZnSnO_3_ and ZnO layers. To elucidate the charge distribution at the interface, the charge density (ρ) and charge density difference (Δρ) at equilibrium interlayer distances were analyzed. Figure 4d reveals that the charge density of the ZnSnO_3_ interfaces (yellow) is higher than that of the ZnO interfaces (blue). In Fig. 4f (above), the charge redistribution at the interfaces is shown, showing an accumulation of electrons in ZnSnO_3_ (indicated by yellow isosurfaces) and a depletion of electrons in ZnO (indicated by blue isosurfaces). This leads to ZnSnO_3_ acquiring a higher negative charge and ZnO a higher positive charge, establishing an internal electric field directed from ZnO to ZnSnO_3_. Figure 4f (below) displays the planar-averaged charge density difference along the Z direction, highlighting significant charge density variations concentrated at the equilibrium interfaces. These observations confirm, through calculations, the occurrence of chemical adsorption and the alignment of energy levels at the ZnO/ZnSnO_3_ interfaces [37]. Further calculations of the charge density differences were conducted to examine electronic transfer during the sensing reactivity. Figure 4f displays blue and yellow lobes representing charge depletion and accumulation during 2-undecanone adsorption, respectively. The transfer of 1.4 electrons from 2-undecanone to the ZnO/ZnSnO_3_ surface aligns with the charge distribution observed in the two-dimensional plane of the heterojunctions (Fig. 4e). Above all, ZnO/ZnSnO_3_ heterojunctions with dual-mesoporous structures exhibit exceptional gas-sensing performance towards 2-undecanone at room temperature. This performance is attributed to the formation of heterostructures facilitated by the nanoparticles composed of Zn and Sn species. Additionally, the combination of SPPS and the regulation of mesoporous structures further enhances gas-sensing reactivity.

**Fig. 5:**
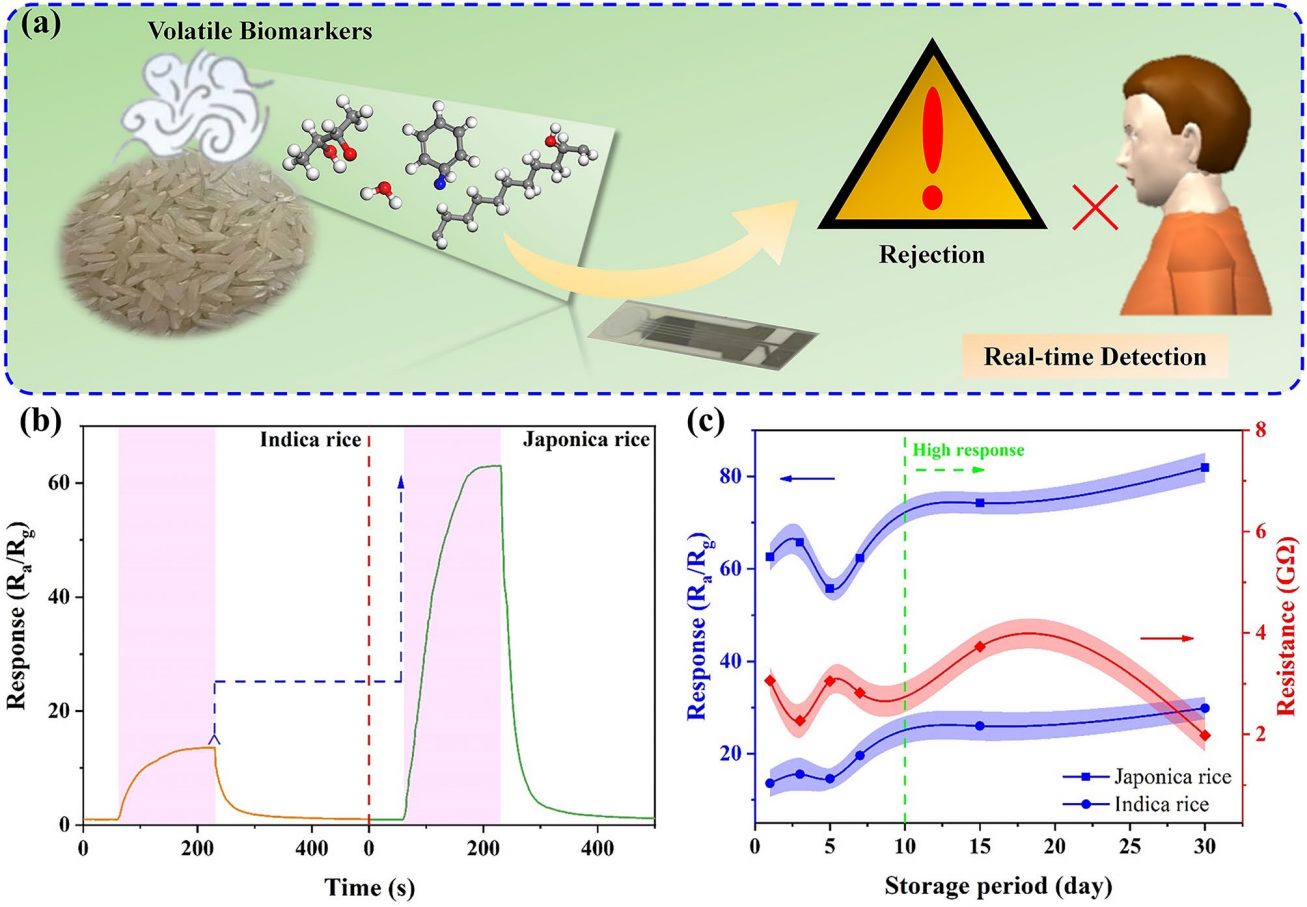
**a** Schematic diagram of the sensor utilized in the real-time detection of volatile compounds generated from rice aging. **b** Responses measured under exposure to volatile gases generated from 30 g Japonica and Indica rice aging. **c** Response and baseline resistance of the sensor towards volatile compounds generated from Japonica rice stored in different periods (1, 3, 5, 7, 15, and 30 days). (The error bar in Fig. 5c is the standard deviation of the three independent measurements)

To evaluate the potential application of the fabricated gas sensor in assessing rice quality, the changes in the electrical resistance of the H3-based gas sensor were measured in response to volatile gases emitted by two varieties of rice stored for different periods (1, 3, 5, 7, 15, and 30 days), as shown in Fig. 5a. In Fig. 5b, the sensor’s response to volatile compounds from Japonica rice is 62.9, roughly 4.6 times greater than that for Indica rice. This suggests that the developed gas sensor could serve as a detection unit in an E-nose for distinguishing Japonica rice from Indica rice. Additionally, based on the changes in resistance, the corresponding sensitivity and *R*_*a*_ values were obtained. Figure 5c shows that there is no significant change in both sensitivity and *R*_*a*_ when the storage period is less than 10 days, which can be attributed to the slow aging process of Japonica and Indica rice at room temperature. The slight fluctuations observed may be due to varying testing conditions, such as humidity, temperature, and pressure, which can significantly influence the results [2, 11]. However, sensitivity slightly increases when the storage period exceeds 15 days, particularly after 30 days, demonstrating that the aging process in Japonica rice is gradually occurring. In conclusion, these experiments suggest that the H3-based gas sensor shows promise for use in developing a high-performance E-nose for assessing rice quality during the aging process, especially in identifying adulteration in different rice varieties.

## Conclusions

Perovskite-structured zinc stannate heterojunctions with dual-mesoporous structures and varying concentrations of oxygen vacancies were directly manufactured by solution precursor plasma spray (SPPS). These heterojunctions were engineered to develop energy-efficient and high-performance gas-sensing devices employed for detecting the characteristic biomarker, 2-undecanone, during rice aging. Characterization of the crystal structures confirms the formation of n–n heterojunctions between ZnO and ZnSnO_3_ interfaces during the spraying process, utilizing precursors with a molar ratio of 1:1. The microstructures of the gas-sensing coatings were rationally designed by controlling a key parameter (hydrogen flow). Notably, the sensor fabricated with low hydrogen flow (H1 and H3) exhibits dual-mesoporous structures, while H5 displays a higher concentration of oxygen vacancies. When employed as gas sensors, H3 demonstrates superior room temperature gas-sensing properties, including higher sensitivity (11.03), a short response time (~ 20 s), and a low theoretical limit of detection (0.4 ppm) towards 2-undecanone, compared to the other two samples. DFT calculations, including band structures and charge density differences, reveal that the strong adsorption of 2-undecanone alters the electrical structures of ZnO/ZnSnO_3_ heterojunctions, contributing to improved room temperature gas-sensing properties. Furthermore, for potential application exploitation, the H3-based gas sensor was applied to monitor the volatile compounds generated during rice aging. The sensor exhibits a high sensitivity of 62.9 towards VOCs from Japonica rice, which is approximately 4.5 times higher than that of Indica rice. This suggests that the sensor based on this semiconductor could be utilized as a detection unit in an E-nose for rapidly identifying adulteration in other rice varieties. In summary, this strategy can be extended to fabricate heterostructured metal oxides with highly concentrated oxygen vacancies and dual-mesoporous structures by adjusting the evaporation rate of the precursors. It holds great potential for applications in developing high-performance E-noses for evaluating agricultural food safety. Importantly, this study opens new avenues for unexplored applications of metal oxide heterojunctions and promotes the development of flexible gas sensors based on this semiconductor.

## Supplementary Information

Below is the link to the electronic supplementary material.

Supplementary file1 (DOCX 3626 KB)
