# Supplementary material for: Ultrasensitive Chemiresistive Gas Sensors Based on Dual-Mesoporous Zinc Stannate Composites for Room Temperature Rice Quality Monitoring
Source: Nanomicro Lett. 2025 Jan 24;17:115. doi: 10.1007/s40820-024-01645-5 (PMC11759721; doi:10.1007/s40820-024-01645-5)
Supplement: Supplementary file 1 — Supplementary file1 (DOCX 3626 KB) [file 40820_2024_1645_MOESM1_ESM.docx]

Supplementary Information for

**Ultrasensitive Chemiresistive Gas Sensors Based on Dual-Mesoporous Zinc Stannate Composites for Room-Temperature Rice Quality Monitoring**

Jinyong Xu^1^, Xuxiong Fan^1^, Kaichun Xu^1, 2^, Kaidi Wu^1^, Hanlin Liao^2^, and Chao Zhang^1,^ *

^1^ College of Mechanical Engineering, Yangzhou University, Yangzhou University 225127, Jiangsu Province P. R. China

^2^ ICB UMR 6303, CNRS, Univ. Bourgogne Franche-Comté, UTBM, 90010 Belfort, France

*Corresponding author. E-mail: [zhangc@yzu.edu.cn](mailto:zhangc@yzu.edu.cn) (Chao Zhang)

**S1 Experimental Section**

**S1.1 Chemicals and materials**

Zinc nitrate hydrate (Zn(NO_3_)_2_∙6H_2_O, 98.0%) and Tin (IV) chloride pentahydrate (SnCl_4_·5H_2_O, 98.0%) were purchased from Sigma Aldrich Chemie GmbH (Germany). 2-Undecanone liquids (99.0%) were bought from Shanghai Aladdin Biochemical Technology Co., Ltd. Standard synthetic air (79%N_2_+21%O_2_) was purchased from Nanjing Special Gas Factory Co., Ltd (Nanjing, China). All chemicals and materials were of analytical grade and used without any further purification. The deionized water (18 MΩ/cm) used in all experiments was prepared by passing through an ultra-pure purification system (Aqua Solutions).

**S1.2 Structure and dimension of the sensor device**

The Pt layer was screen-printed on the alumina substrate (6*30 mm) (Figure S2), composed of a heating and measuring unit. The gap between the gear-shaping electrodes was 0.42 mm. The gas sensing measurement was carried out by the four-channel gas sensing testing instrument (Wuhan Huachuang Ruike Technology Co., Ltd., China), which can show real-time electrical resistance signals.

**S1.3 Methods of detecting 2-undecanone concentration**

The gas-sensing experiments were conducted under controlled laboratory conditions with a temperature of 25±2 ℃ and a relative humidity of 30%±10%, maintained using air conditioning. As illustrated in Figure S2, the flow (x) of 2-undecanone with a concentration of 13 ppm was controlled with one mass flow controller (MFC), the flow (y) of high-purity mixed air (Nanjing Special Gas Factory Co., Ltd.) was controlled with another MFC. The theoretical concentrations (Unit: ppm) were defined as: (theoretical concentration*x)/(x+y), by controlling the flow separately. Except in the experiment to investigate the effect of relative humidity (RH), the flow (z) of high-purity mixed air was controlled with another MFC and bubbled via the deionized water. The RH (Unit: %) was defined as z/(x+y+z)*%.

**S1.4 Method used to determine the electrical resistance**

The data logger supplies a steady voltage of 12 V (*V_EX_*). This voltage is distributed between the reference resistor (*R_ref_*), which has a known and constant resistance, and the IDA-CR, whose resistance changes according to the detected 2-undecanone concentration. The voltage is relatively distributed according to the resistance of the two circuit components. Therefore, given the voltage between *V_EX_* and ground (GND), the voltage between *V_meas_* and GND is measurable. From their ratio, it is possible to calculate IDA-CR resistance, as described in Equation S1.

*V_means_/V_EX_=R_CR_/(R_CR_+R_ref_) → R_CR_=(R_ref_·V_means_)*$\frac{V_{\mathrm{meas}}}{V_{\mathrm{EX}}}=\frac{R_{\mathrm{CR}}}{R_{\mathrm{CR}}+R_{\mathrm{ref}}} \to R_{\mathrm{CR}}=\frac{R_{\mathrm{ref}}\cdot(\frac{V_{\mathrm{meas}}}{V_{\mathrm{EX}}})}{1-(\frac{V_{\mathrm{meas}}}{V_{\mathrm{EX}}})}$*/(V_EX_-V_means_)* (S1)

**S1.5 Theoretical calculation**

All calculations were carried out based on density functional theory (DFT) as implemented in the Vienna ab initio simulation package (VASP) with exchange-correlation functional of generalized gradient approximation (GGA) using the Perdew-Burke-Emzerhof (PBE) method. A grid of 1×1×1 Monkhorst-Pack K-points was used for the structural relaxation. A vacuum layer of 15 Å was adopted in the direction perpendicular to the monolayer surface to avoid the interactions between periodic slabs. Grimme’s DFT-D3 methodology was chosen to describe the dispersion interactions and considered the valence electrons using a plane wave basis expansion with an energy cutoff of 450 eV. Partial occupancies of the Kohn-Sham orbitals were allowed utilizing the Gaussian smearing method and a width of 0.05 eV. The self-consistent calculations apply a convergence energy threshold of 10^-5^ eV. The adsorption ability was evaluated by comparing the adsorption energy (*E_ads_*) calculated based on Equation S2.

*E_ads_=E_gas/surface_-E_surface_-E_gas_*  (S2)

Where *E_gas/surface_*, *E_surface,_* and *E_gas_* are the energy of adsorbate gas adsorbed on the surface, clean surface, and isolated gas molecules in a cubic periodic box, respectively.

In this work, according to the analysis of the XRD patterns shown in Figure 2a, ZnSnO₃ is ilmenite-like structured and crystallizes in the trigonal R3c space group. ZnO is Wurtzite structured and crystallizes in the hexagonal P63mc space group. The relative concentration of oxygen vacancy obtained from the corresponding XPS analysis was also considered in the establishment of the heterojunction model. Their visualized structures are vividly illustrated in Figs. S14 and S17.

**S2 Supplementary Figures and Tables**


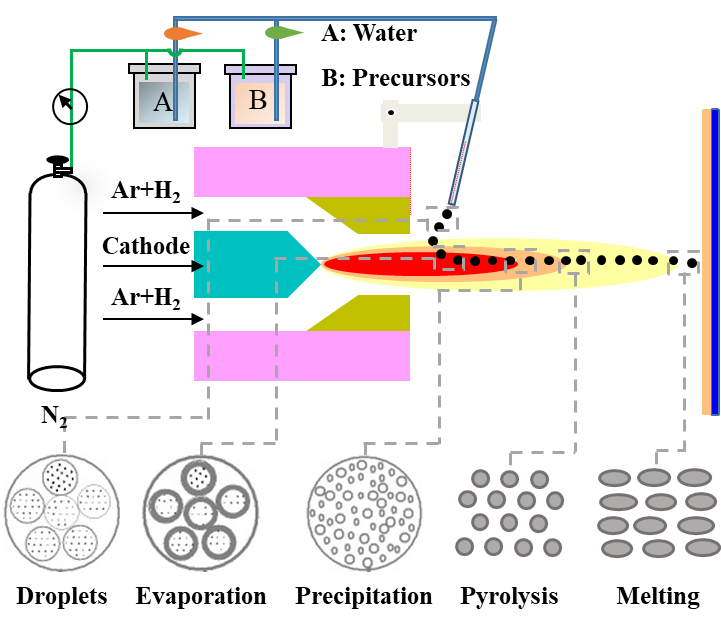


**Fig. S1** Schematic illustration of the preparation and the evolution of the gas-sensing coatings deposited by SPPS


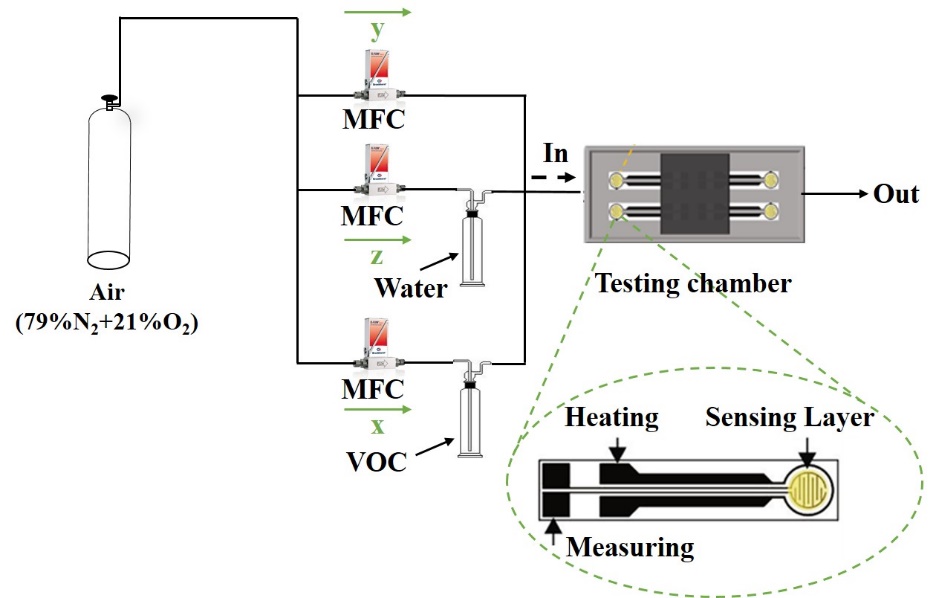


**Fig. S2** Schematic illustration of the gas sensing testing system utilized in the gas-sensing experiment


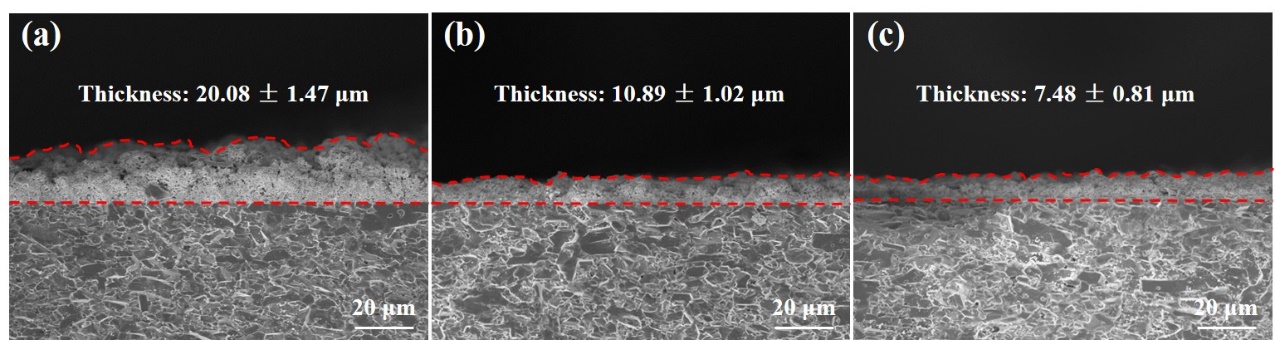


**Fig. S3** Cross-sectional profiles of (a) H1, (b) H3, and (c) H5 directly deposited by SPPS


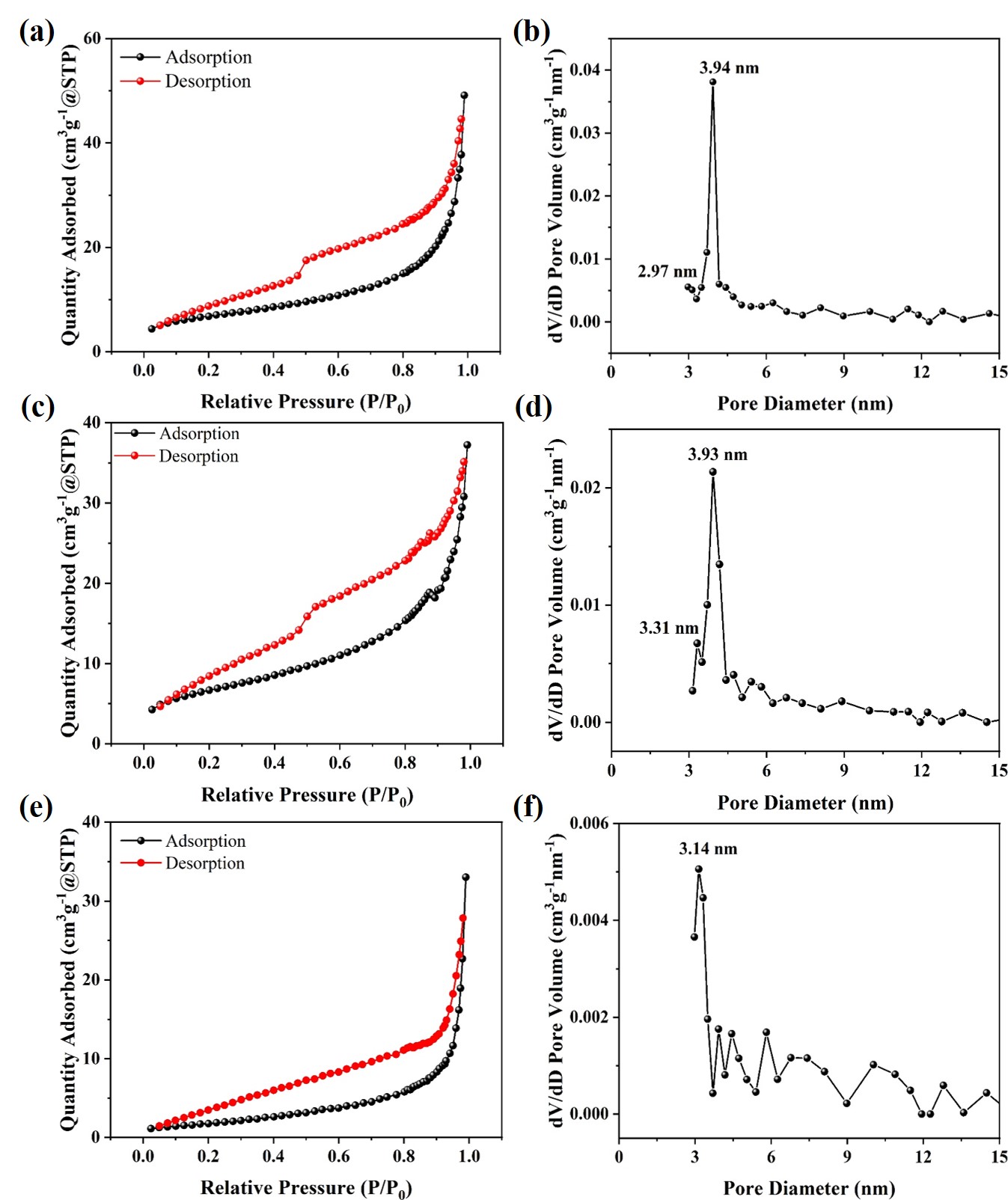


**Fig. S4** Nitrogen adsorption-desorption isotherms and corresponding pore size distribution curves of (**a, b**) H1, (**c, d**) H3, and (**e, f**) H5


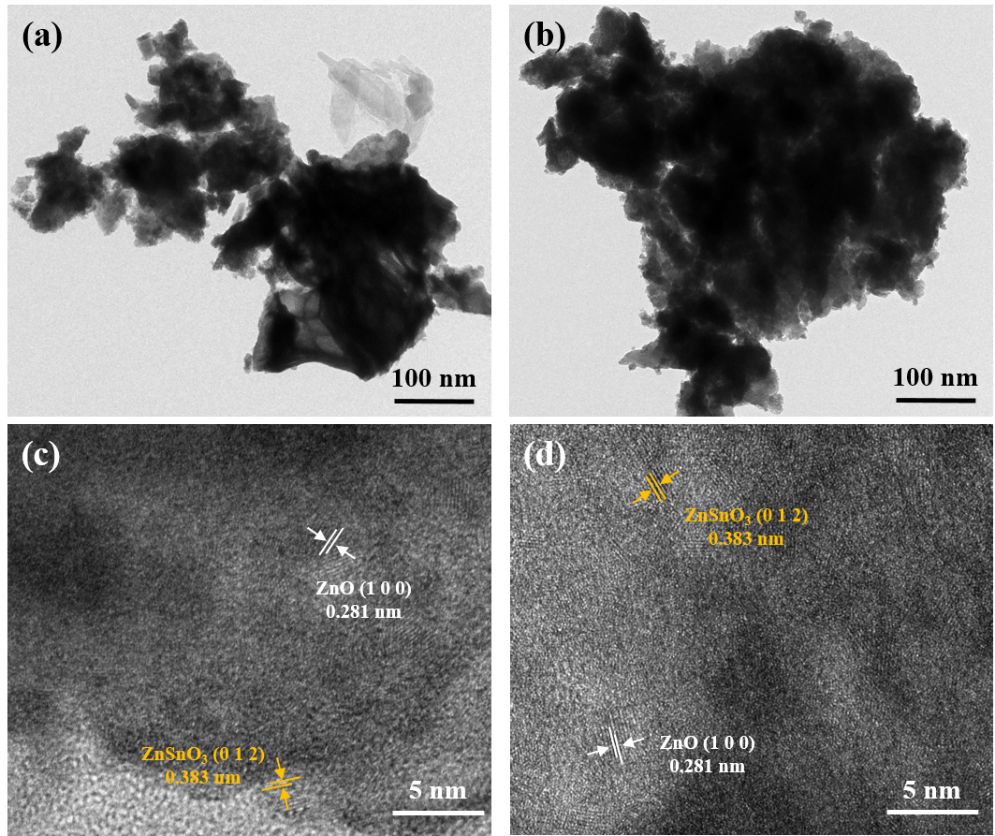


**Fig. S5** TEM and HRTEM images of (**a, c**) H1 and (**b, d**) H5


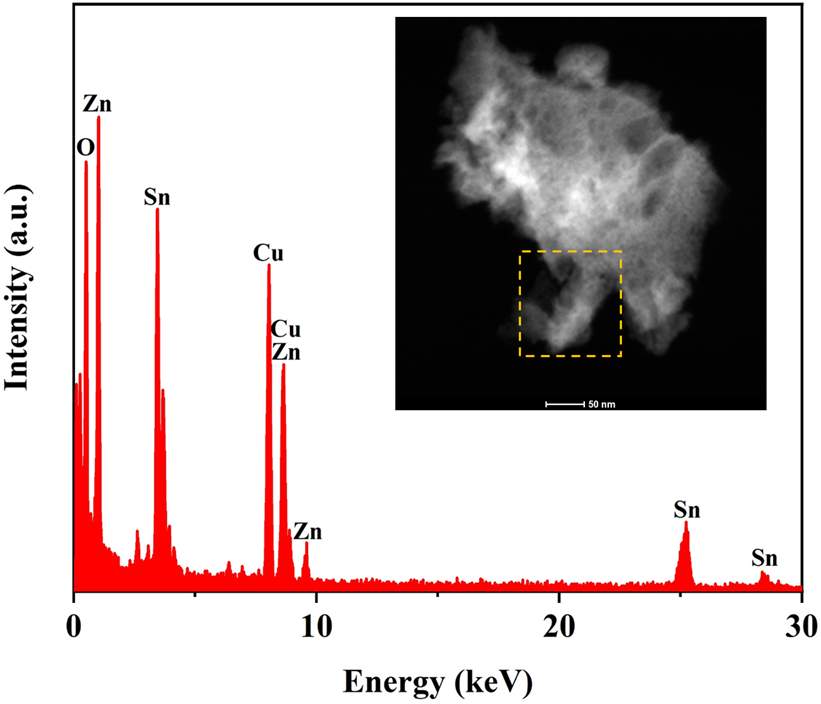


**Fig. S6** Energy dispersive spectrum (EDS) of ZnO/ZnSnO_3_ nanoscale heterojunctions fabricated with the hydrogen flow of 3 L/min


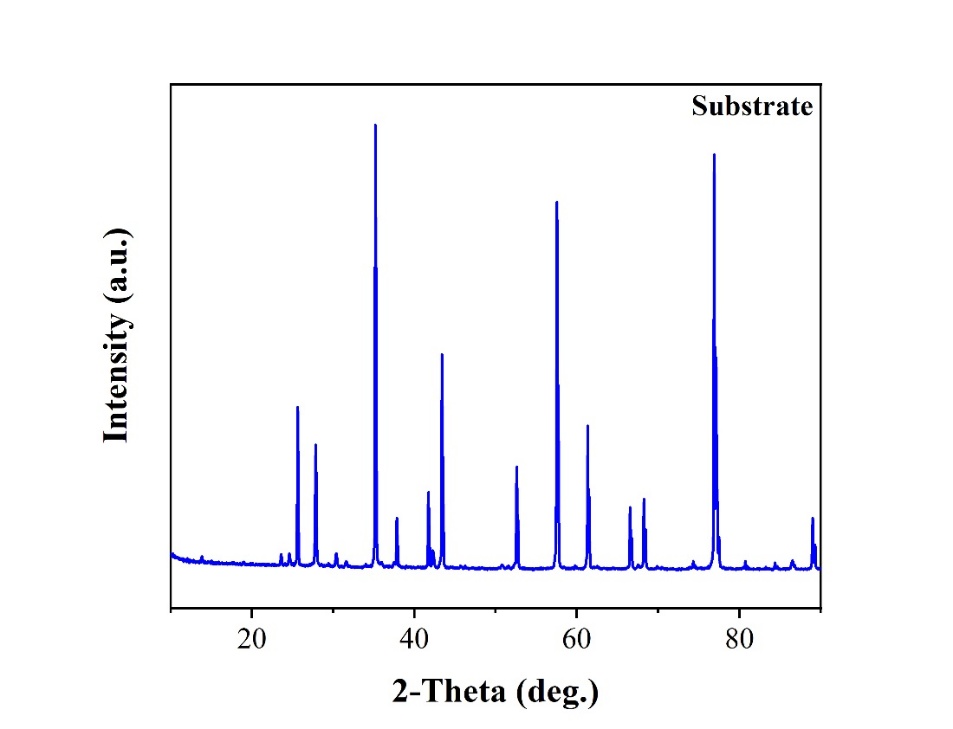


**Fig. S7** XRD pattern of the substrate used in this work


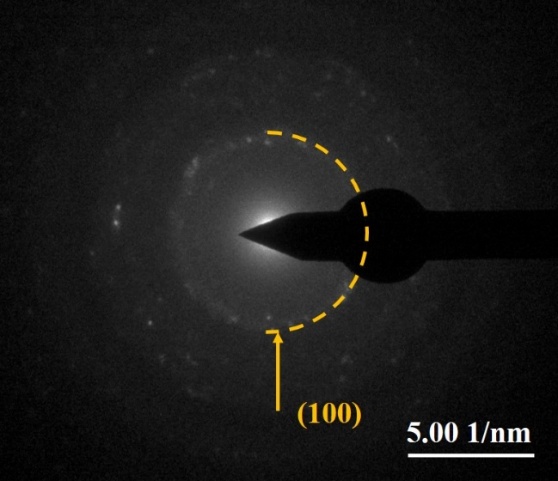


**Fig. S8** Selected-area electron diffraction (SAED) pattern of ZnO/ZnSnO_3_ nanoscale heterojunctions fabricated with the hydrogen flow of 3 L/min


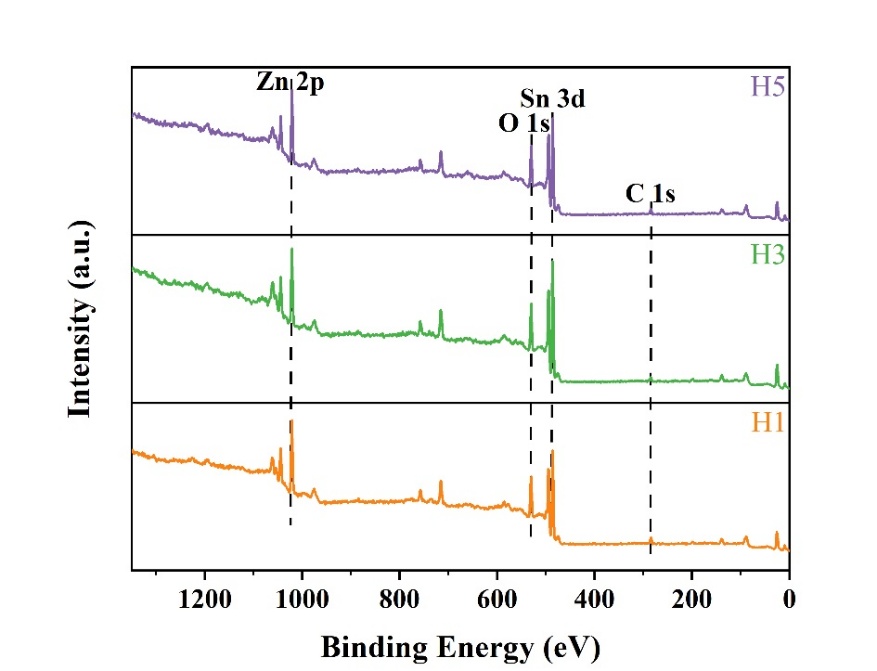


**Fig. S9** XPS analysis on survey spectra of H1, H3, and H5


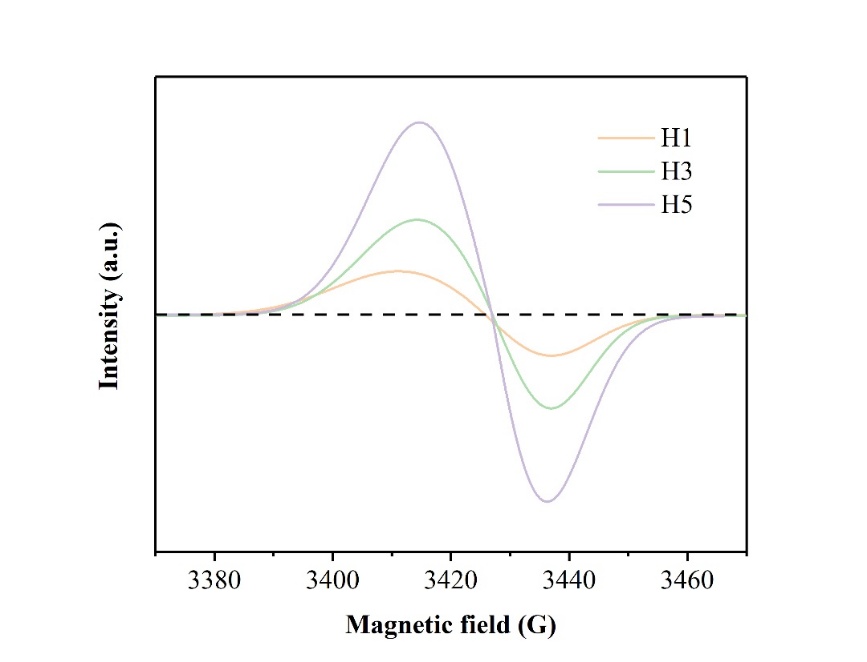


**Fig. S10** EPR spectra of all samples


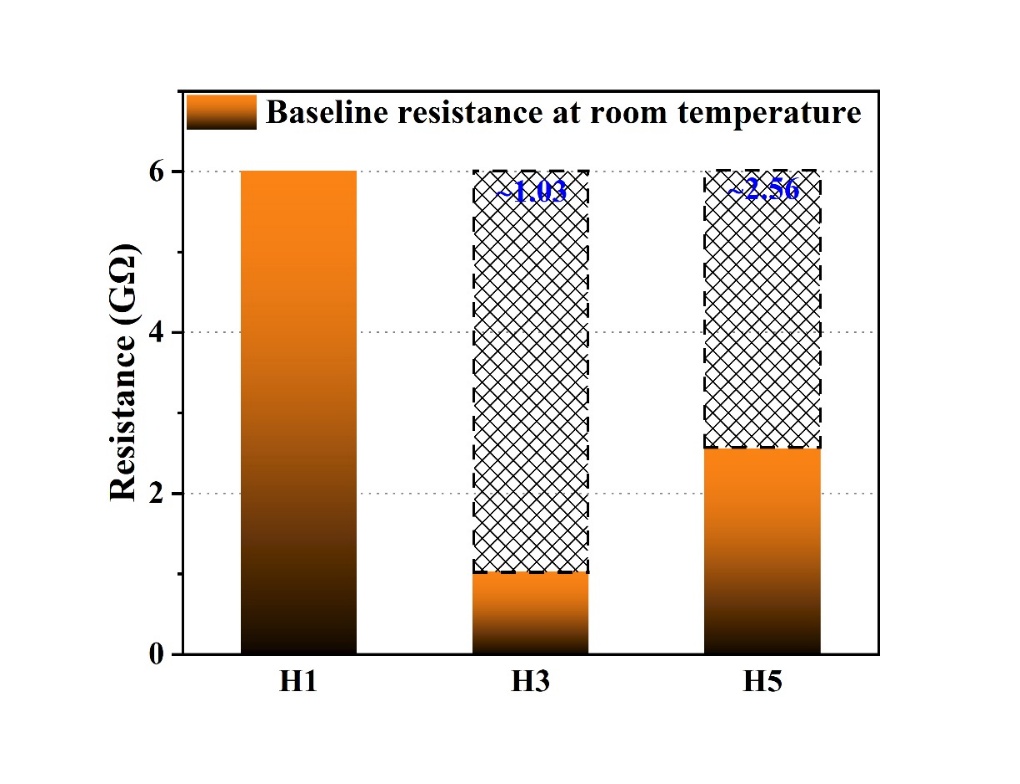


**Fig. S11** Baseline resistance of H1, H3, and H5-based gas sensors towards the purified air at room temperature


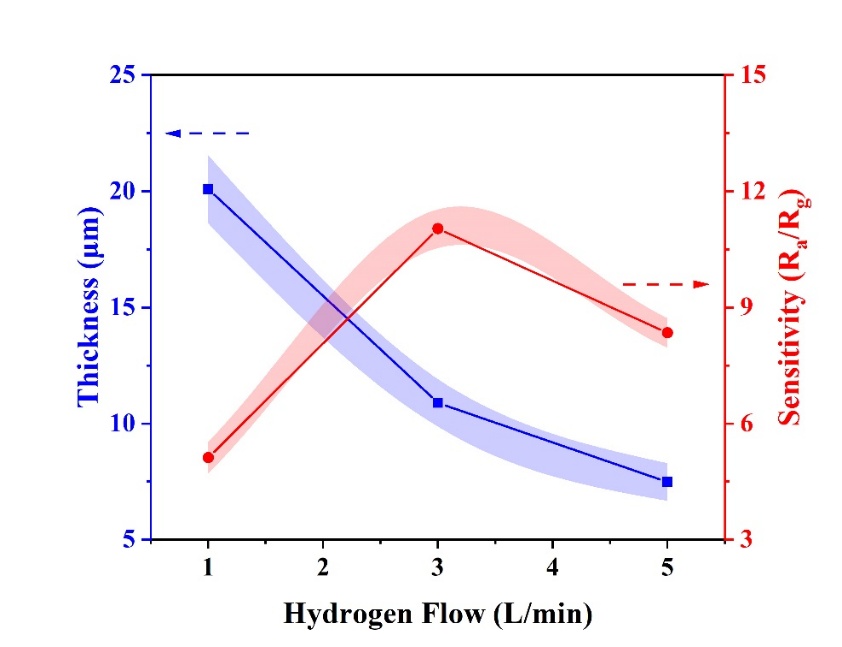


**Fig. S12** Relationship between sensitivity and thickness of all samples deposited by SPPS


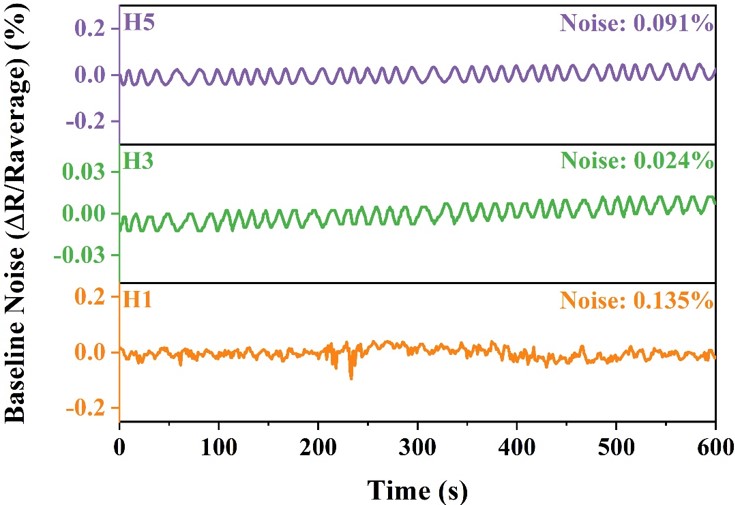


**Fig. S13** Baseline noise of the sensors during purified air (79%N_2_+21%O_2_) exposure


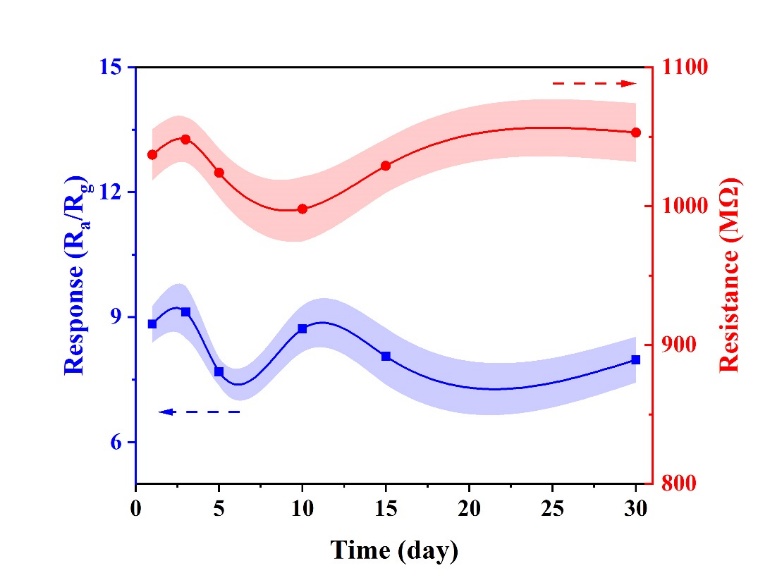


**Fig. S14** Long-term stability including sensitivity and baseline resistance (*R_a_*) of H3 towards 10 ppm 2-Undecanone at room temperature


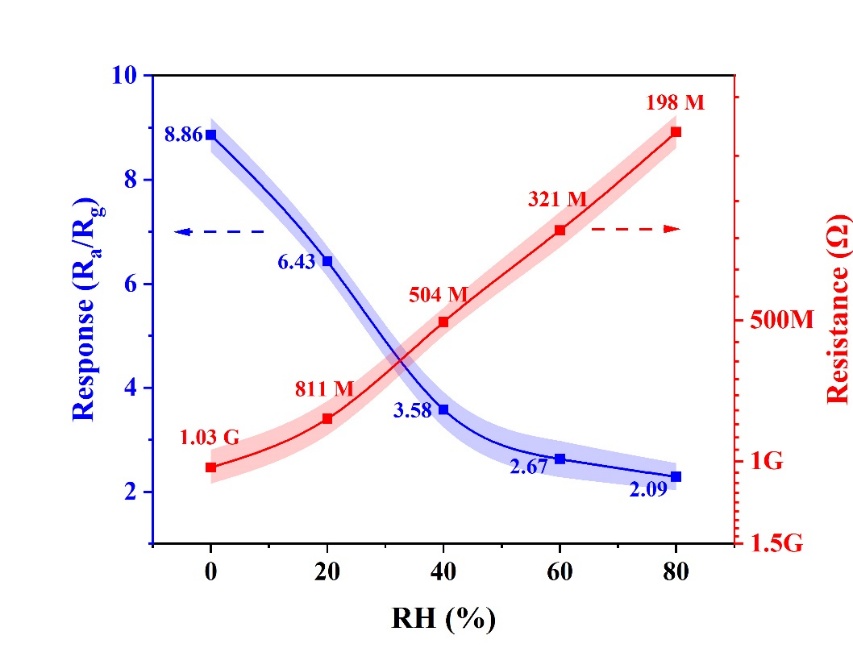


**Fig. S15** Sensor responses and its corresponding baseline resistance (*R_a_*) of H3 towards 10 ppm 2-Undecanone under various relative humidity conditions (0%, 20%, 40%, 60%, and 80%RH)


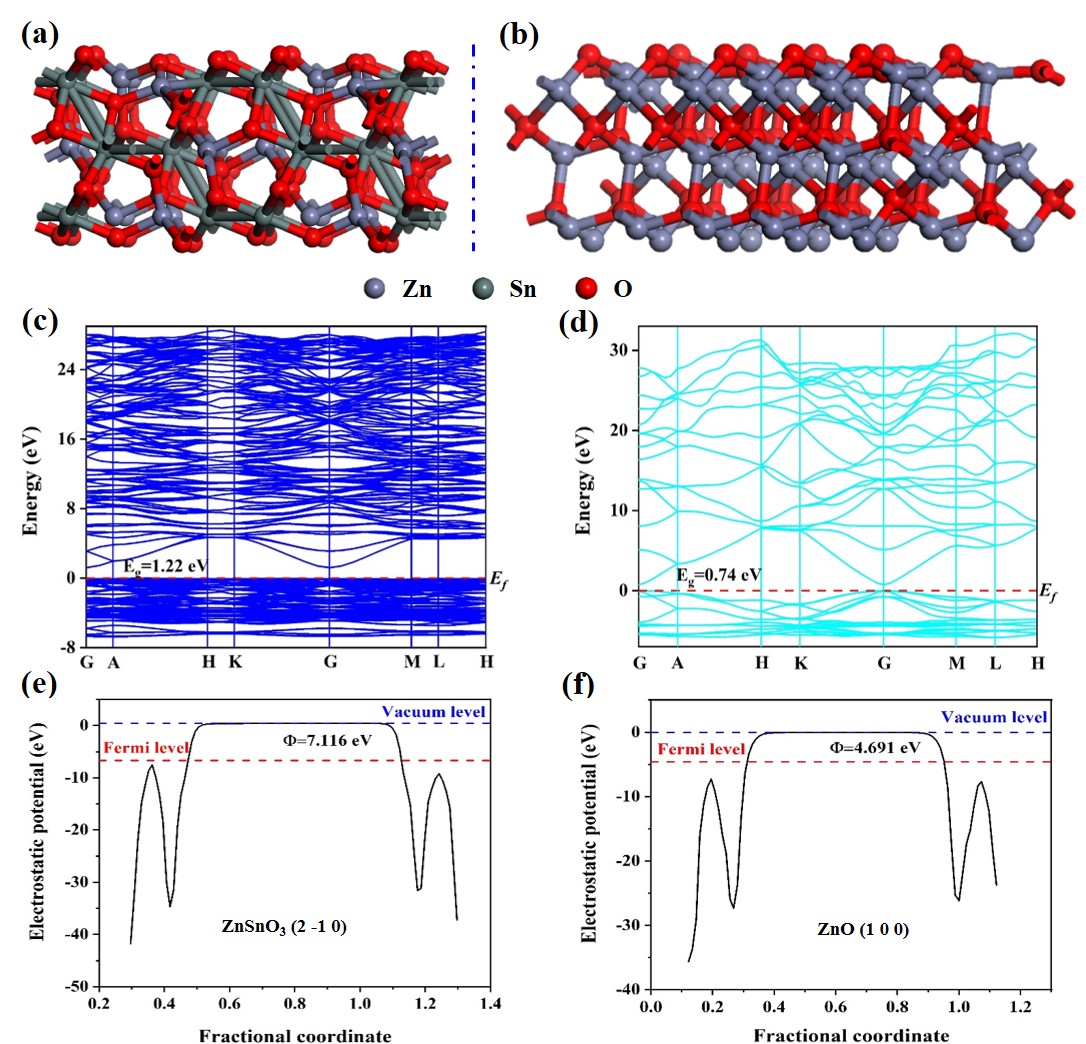


**Fig. S16** The visualized structures, the calculated band structures and the work functions of **a, c, e** ZnSnO_3_ and **b, d, f** ZnO obtained from the DFT calculations.


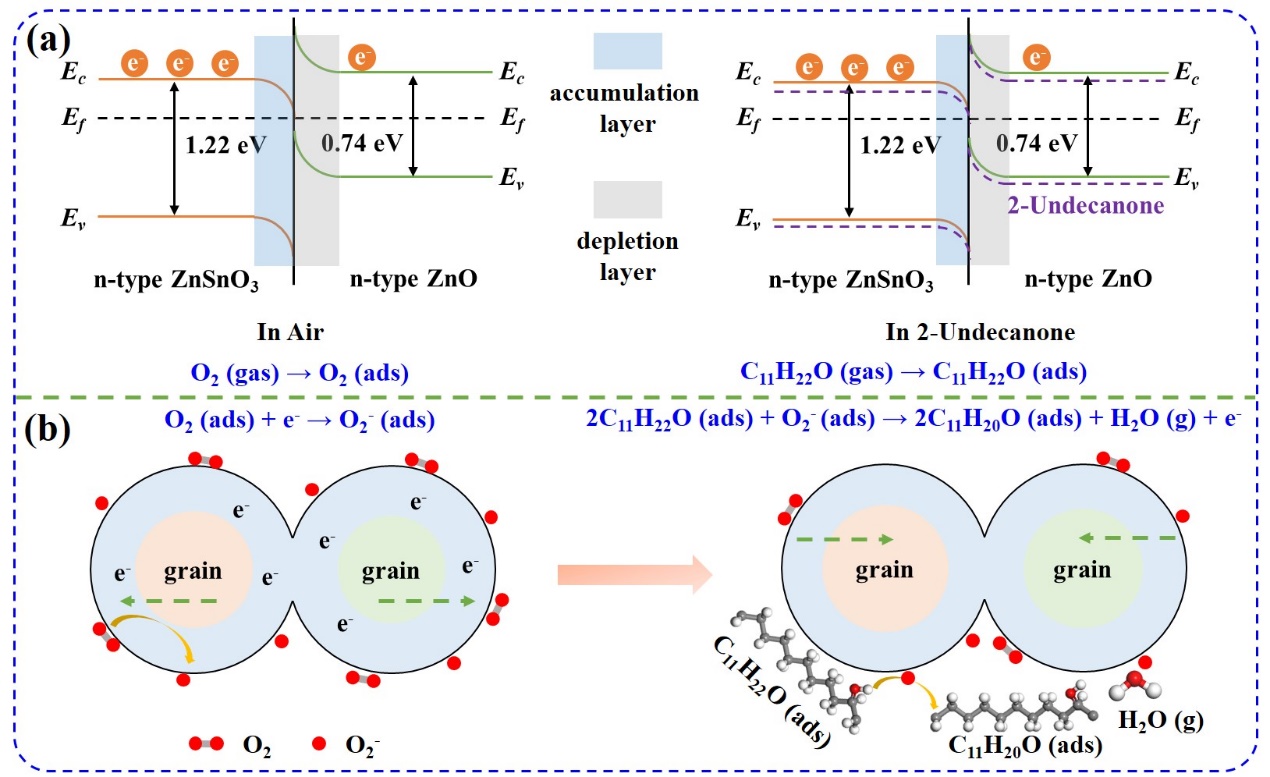


**Fig. S17** Room-temperature gas-sensing mechanisms of zinc stannate heterojunctions towards 2-Undecaone. **a** The band alignment and energy diagram after contact. **b** Schematic diagrams for the adsorption of 2-Undecaone on the surface of sensing materials (*E_c_*: conduction band bottom; *E_f_*: Fermi level; *E_v_*: valence band top)


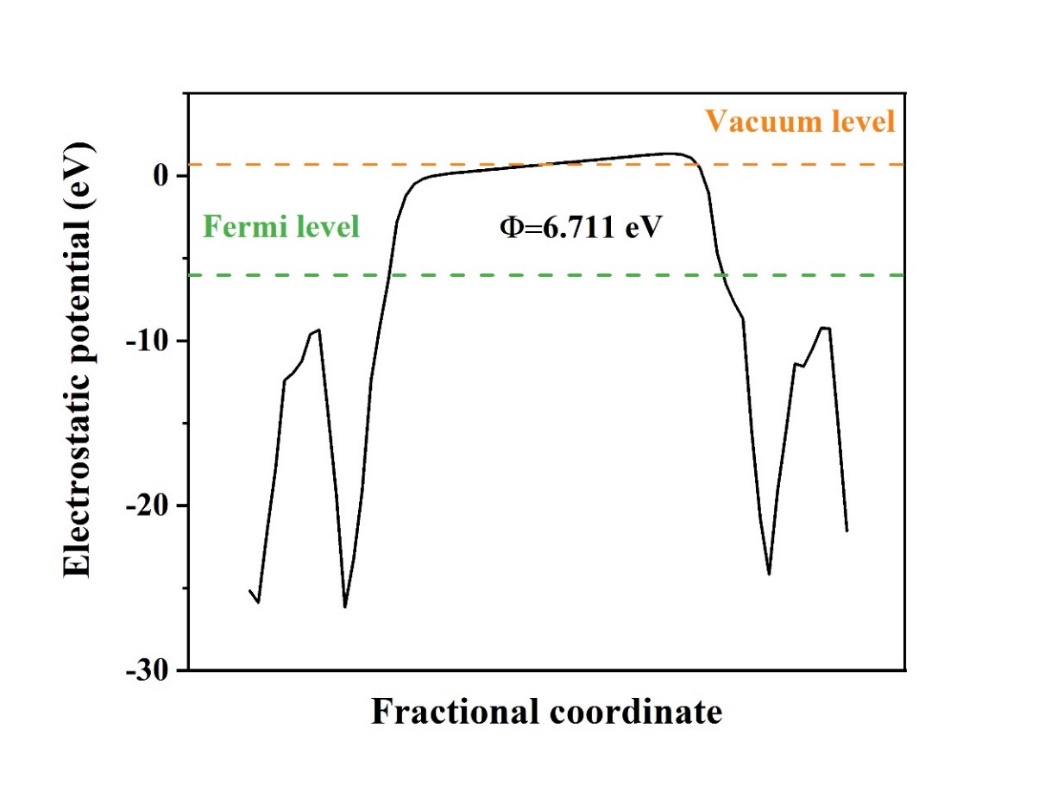


**Fig. S18** The calculated work function of zinc stannate heterojunctions (ZnO/ZnSnO_3_)


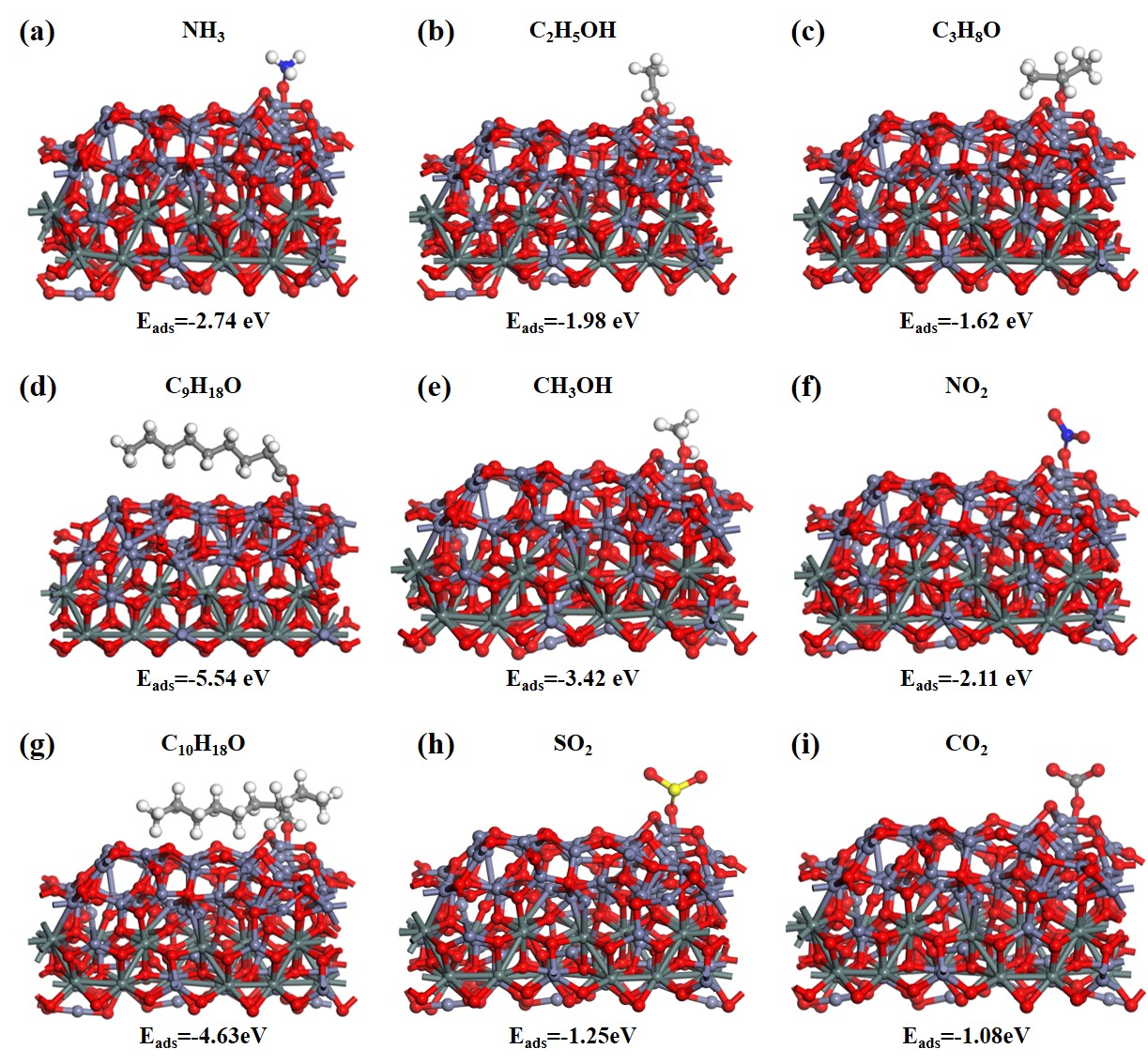


**Fig. S19** The analysis of the adsorption structure with geometric binding configuration for the model of nine gases on the zinc stannate heterojunctions

**Table S1** Spraying parameters for ZnO/ZnSnO_3_ nanoscale heterojunctions by SPPS

| Parameter/Unit | Value |
| --- | --- |
| Current/A | 580 |
| Ar flow/L∙min^-1^ | 35 |
| H_2_ flow/L∙min^-1^ | 1, 3, 5 |
| Spray distance/mm | 90 |
| Solution concentration/M | 0.3 |
| Substrate preheating temperature/℃ | 400 |
| Solution flow rate/mL∙min^-1^ | 28 |

**Table S2** Average crystallite size of all samples

|  | H1 | H2 | H3 |
| --- | --- | --- | --- |
| Size/nm | 7.53 | 10.36 | 14.43 |

**Table S3** Textural properties of the samples deposited by SPPS

| Sample | BET surface area/m^2^∙g^-1^ | Pore diameter/nm | Pore volume/cm^3^∙g^-1^ |
| --- | --- | --- | --- |
| H1 | 24.500 | 2.97, 3.94 | 0.073 |
| H3 | 24.086 | 3.31, 3.93 | 0.057 |
| H5 | 7.107 | 3.14 | 0.051 |

**Table S4** The gas-sensing performance of all samples towards 13 ppm 2-Undecanone at 25 ± 1 ℃, 30 ± 10%RH

| Material | Hydrogen/L·min^-1^ | Sensitivity (R_a_/R_g_) | Response time (s) | Recovery time (s) | Linear coefficient |
| --- | --- | --- | --- | --- | --- |
| ZnO | 3 | 3.47 | 29 | 38 | - |
| SnO_2_ | 3 | 2.89 | 30 | 52 | - |
| ZnO/ZnSnO_3_ | 1 | 5.12 | 33 | 28 | 0.995 |
|  | 3 | 11.03 | 21 | 34 | 0.981 |
|  | 5 | 8.34 | 24 | 48 | 0.984 |

**Table S5** Detailed parameters utilized for DFT calculations

| Model | E_cut_/eV | K-point | (h k l) |
| --- | --- | --- | --- |
| ZnO | 500 | 13×13×7 | (1 0 1) |
| ZnSnO_3_ | 450 | 5×5×2 | (2 -1 0) |
